# Group Guided Fused Laplacian Sparse Group Lasso for Modeling Alzheimer's Disease Progression

**DOI:** 10.1155/2020/4036560

**Published:** 2020-02-20

**Authors:** Xiaoli Liu, Jianzhong Wang, Fulong Ren, Jun Kong

**Affiliations:** ^1^College of Humanities and Sciences, Northeast Normal University, Changchun, China; ^2^Education AI, College of Information Science and Technology, Northeast Normal University, Changchun, China; ^3^Key Laboratory of Applied Statistics of MOE, Northeast Normal University, Changchun, China; ^4^Key Laboratory of Medical Image Computing of Ministry of Education, Northeastern University, Shenyang, China

## Abstract

As the largest cause of dementia, Alzheimer's disease (AD) has brought serious burdens to patients and their families, mostly in the financial, psychological, and emotional aspects. In order to assess the progression of AD and develop new treatment methods for the disease, it is essential to infer the trajectories of patients' cognitive performance over time to identify biomarkers that connect the patterns of brain atrophy and AD progression. In this article, a structured regularized regression approach termed group guided fused Laplacian sparse group Lasso (GFL-SGL) is proposed to infer disease progression by considering multiple prediction of the same cognitive scores at different time points (longitudinal analysis). The proposed GFL-SGL simultaneously exploits the interrelated structures within the MRI features and among the tasks with sparse group Lasso (SGL) norm and presents a novel group guided fused Laplacian (GFL) regularization. This combination effectively incorporates both the relatedness among multiple longitudinal time points with a general weighted (undirected) dependency graphs and useful inherent group structure in features. Furthermore, an alternating direction method of multipliers- (ADMM-) based algorithm is also derived to optimize the nonsmooth objective function of the proposed approach. Experiments on the dataset from Alzheimer's Disease Neuroimaging Initiative (ADNI) show that the proposed GFL-SGL outperformed some other state-of-the-art algorithms and effectively fused the multimodality data. The compact sets of cognition-relevant imaging biomarkers identified by our approach are consistent with the results of clinical studies.

## 1. Introduction

Alzheimer's disease (AD) is a chronic neurodegenerative disease, which mainly affects memory function, and its progress ultimately culminates in a state of dementia where all cognitive functions are affected. Therefore, AD is a devastating disease for those who are affected and presents a major burden to caretakers and society. According to reports conducted by the Alzheimer's Disease Neuroimaging Initiative (ADNI), the worldwide prevalence of AD would be 131.5 million by the year 2050, which is nearly three times as much as the number in 2016 (i.e., 46.8 million) [1]. Moreover, the total worldwide cost of dementia caused by AD is about 818 billion US dollars, and it will become a trillion dollar disease by 2018 [1].

According to some researches, there exists strong connection between patterns of brain atrophy and AD progression [2, 3]. Thus, it is important to utilize some measurements to assess the patients' cognitive characterization so that the development of AD can be monitored [4, 5]. In the clinical field, the criteria such as Mini Mental State Examination (MMSE) and Alzheimer's Disease Assessment Scale cognitive subscale (ADAS-Cog) have been widely applied to evaluate the cognitive status of patients for diagnosis of probable AD. However, the results of these clinical criteria may be affected by demographic factors and insensitive to progressive changes occurring with severe Alzheimer's disease [6]. Furthermore, accurate diagnosis based on these criteria also depends on a doctor's expertise. Recently, some machine learning-based techniques have been employed in AD research. Compared with the clinical criteria, these machine learning approaches are always data-oriented. That is, they seek to infer patient's cognitive abilities and track the disease progression of AD from biomarkers of neuroimaging data such as magnetic resonance imaging (MRI) and positron emission tomography (PET).

Regression-based models could explore the relationship between cognitive abilities of patients, and some valuable factors that may cause AD or affect disease development were widely applied for AD analysis field. Some early studies establish regression models for different cognitive scores or the same cognitive score over time independently. However, researchers have found that there exist inherent correlations among different cognitive scores or the same cognitive score over time, largely because the underlying pathology is the same and there is a clear pattern in disease progression over time [7–10]. To achieve a more accurate predictive ability, multitask learning (MTL) was introduced for AD analysis to learn all of the models jointly rather than separately [11]. In many studies, it has been proven that MTL could obtain better generalization performance than the approaches learning each task individually [12, 13]. An intuitive way to characterize the relationships among multiple tasks is to assume that all tasks are related and their respective models are similar to each other. In [14], Zhang et al. considered regression models of different targets (such as MMSE and ADAS-Cog) as a multitask learning problem. In their method, all regression models are constrained to share a common set of features so that the relationship among different tasks can be captured. Wan et al. [15] proposed an approach called sparse Bayesian multitask learning. In this approach, the correlation structure among tasks is adaptively learnt through constraining the coefficient vectors of the regression models to be similar. In [16], the sparse group Lasso (SGL) method was also adopted to consider two-level hierarchy with feature-level and group-level sparsity and parameter coupling across tasks.

Besides, there also exist some studies which focused on analyzing longitudinal data of AD by MTL. That is, the aim of each task is to model a given cognitive score at a given time step, and different tasks are utilized to model different time steps for the same cognitive score. For AD, longitudinal data usually consist of measurements at a starting time point (*t* = 0), after 6 months (*t* = 6), after 12 months (*t* = 12), after 24 months (*t* = 24), and so on usually up to 48 months (*t* = 48). Zhou et al. employed MTL algorithm for longitudinal data analysis of AD [9]. In this work, we develop temporal group Lasso (TGL) regularization to capture the relatedness of multiple tasks. However, since the TGL enforces different regression models to select the same features at all time steps, the temporal patterns and variability of the biomarkers during disease progression may be ignored. In order to handle this issue, an MTL algorithm based on convex fused sparse group Lasso (cFSGL) was proposed [10]. Through a sparse group Lasso penalty, cFSGL could select a common set of biomarkers at all time steps and a specific set of biomarkers at different time steps simultaneously. Meanwhile, the fused Lasso penalty in cFSGL also took on the temporal smoothness of the adjacent time steps into consideration [17]. Since cFSGL is nonsmooth, the MTL problem with cFSGL regularization was solved by a variant of the accelerated gradient method.

**Figure 1:**
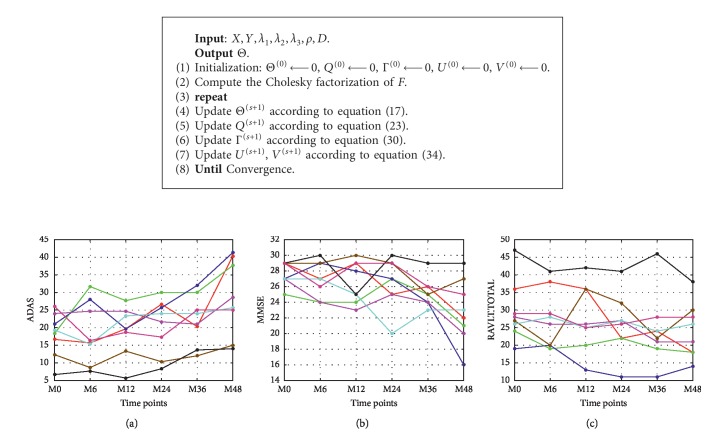
The change patterns of several patients' cognitive scores over the 6 time points: (a) ADAS, (b) MMSE, and (c) RAVLT.TOTAL. The different colors indicate different patients from our dataset.

Though TGL and cFSGL have been successfully implemented for AD analysis, a major limitation of the complex relationships among different time points and the structures within the ROIs are often ignored. Specifically, (1) the fused Lasso in TGL and cFSGL only takes into account the association existing between the two consecutive time points that are likely to skip useful task dependencies beyond the next neighbors. To summarize, in a case where every task (time step) is seen to be a node of a graph, together with the edges determining the task dependencies, cFSGL makes use of a graph where there exist edges between the tasks, *t* and *t*+1,  *t*=1,…, *T* − 1; nonetheless, there do not exist any other edges. Assume that the scores between the two consecutive time points need to be close is quite logical [18]. Nevertheless, concerning medical practice, this supposition is unlikely to stay valid all the time.  Figure 1 sheds light on how not just the real ADAS but also MMSE and RAVLT scores of several subjects from our dataset changed throughout the years. Besides, consistent periods are coupled with sharp falls and tangled with occasional enhancements. It suggests that the longitudinal medical scores are likely to have a more intricate evolution as compared with straightforward linear tendencies with the local temporal relationships [19]. (2) Conversely, concerning MRI data, many MRI attributes are interconnected, in addition to revealing the brain cognitive activities together [20]. In accordance with our data, multiple shape measures (which include volume, area, and thickness) from the same area offer a detailed quantitative assessment of the cortical atrophy, besides tending to be chosen as the collective predictors. Our earlier research work put forward a framework, which made use of the previous knowledge to guide a multitask feature learning framework. This model is an effective approach that uses group information to enforce the intragroup similarity [21]. Thus, exploring and utilizing these interrelated structures is important when finding and selecting important and structurally correlated features together. In our previous work [22], we proposed an algorithm that generalized a fused group Lasso regularization to multitask feature learning to exploit the underlying structures. This method considers a graph structure within tasks by constructing an undirected graph, where the computations are pairwise Pearson correlation coefficients for each pair of tasks. Meanwhile, the method jointly learns a group structure from the image features, which adopts group Lasso for each pair of correlated tasks. Thus, only the relationship between two time points in the graph was considered by the regularization.

For the sake of overcoming these two limitations, a structure regularized regression approach, group guided fused Laplacian sparse group Lasso (GFL-SGL), is proposed in this paper. Our proposed GFL-SGL can exploit commonalities at the feature level, brain region level, and task level simultaneously so as to exactly identify the relevant biomarkers from the current cognitive status and disease progression. Specifically, we designed novel mixed structured sparsity norms, called group guided fused Laplacian (GFL), to capture more general weighted (undirected) dependency graphs among the tasks and ROIs. This regularizer is based on the natural assumption that if some ROIs are important for one time point, it has similar but not identical importance for other time points. To discover such dependent structures among the time points, we employed the graph Laplacian of the task dependency matrix to uncover the relationships among time points. In our work, we consider weighted task dependency graphs based on a Gaussian kernel over the time steps, which yields a fully connected graph with decaying weights. At the same time, through considering the group structure among predictors, group information is incorporated into the regularization by task-specific *G*_2,1_-norm, which leads to enforce the intragroup similarity with group sparse. Besides, by incorporating task-common *G*_2,1_-norm and Lasso penalties into the GFL model, we can better understand the underlying associations of the prediction tasks of the cognitive measures, allowing more stable identification of cognition-relevant imaging markers. Using task-common *G*_2,1_-norm can incorporate multitask and sparse group learning, which learns shared subsets of ROIs for all the tasks. This method has been demonstrated to be an effective approach in our previous study [23]. And Lasso can maintain sparsity between features. The resulting formulation is challenging to solve due to the use of nonsmooth penalties, including the GFL, *G*_2,1_-norm, and Lasso. In this work, we propose an effective ADMM algorithm to tackle the complex nonsmoothness.

We perform extensive experiments using longitudinal data from the ADNI. Five types of cognitive scores are considered. Then, we empirically evaluate the performance of the proposed GFL-SGL methods along with several baseline methods, including ridge regression, Lasso, and the temporal smoothness models TGL [9] and cFSGL [24]. Experimental results indicate that GFL-SGL outperforms both the baselines and the temporal smoothness methods, which demonstrates that incorporating sparse group learning into temporal smoothness and multitask learning can improve predictive performance. Furthermore, based on the GFL-SGL models, stable MRI features and key regions of interest (ROIs) with significant predictive power are identified and discussed. We found that the results corroborate previous studies in neuroscience. Finally, in addition to the MRI features, we use multimodality data including PET, CSF, and demographic information for GFL-SGL as well as temporal smoothness models. While the additional modalities improve the predictive performance of all the models, GFL-SGL continues to significantly outperform other methods.

The rest of the paper is organized as follows. In Section 2, we provide a description of the preliminary methodology: multitask learning (MTL), two types of group Lasso norms, and fused Lasso norm. In Section 3, we present the GFL-SGL model and discuss the details of the ADMM algorithm proposed for the optimization. We present experimental results and evaluate the performance using the MRI data from the ADNI-1 and multimodality data from the ADNI-2 in Section 4. The conclusions are presented in Section 5.

## 2. Preliminary Methodology

### 2.1. Multitask Learning

Take into account multitask learning (MTL) setting having *k* tasks [19, 21]. Suppose that *p* is the number of covariates, which is shared all through each task, *n* indicates the number of samples. Suppose that *X* ∈ *ℝ*^*n*×*p*^ indicates the matrix of covariates, *X* ∈ *ℝ*^*n*×*k*^ implies the matrix of feedbacks with each of the rows that correspond to a sample, and Θ ∈ *ℝ*^*p*×*k*^ suggests the parameter matrix, with column *θ*_.*m*_ ∈ *ℝ*^*p*^ that corresponds to task *m*, *m*=1,…, *k*, and row *θ*_*j*._ ∈ *ℝ*^*k*^ that corresponds to the feature *j*, *j*=1,…, *p*. Besides, the MTL issue can be established to be among the estimations of the parameters based on the appropriate regularized loss function. To associate the imaging markers and the cognitive measures, the MTL model minimizes the objective as follows:

(1) $$\begin{array}{c} \underset{\Theta \in {\mathbb{R}}^{p\times k}}{\min}L\left(Y,X,\Theta \right)+\lambda R\left(\Theta \right), \end{array}$$

where *L*(·) is an indication of the loss function, whereas *R*(·) suggests the regularizer. In the present context, we make an assumption of the loss as a square loss, i.e.,

(2) $$\begin{array}{c} L\left(Y,X,\Theta \right)={\left\|Y-X\Theta \right\|}_{F}^{2}=\overset{n}{\underset{i=1}{\sum }}{\left\|y_{i}-x_{i}\Theta \right\|}_{2}^{2}, \end{array}$$

where *y*_*i*_ ∈ *ℝ*^1×*k*^ and *x*_*i*_ ∈ *ℝ*^1×*p*^ denote the *i*-th rows of *Y* and *X* that correspond to the multitask feedback as well as the covariates for the *i*-th sample. Besides that, we observe the fact that the MTL framework is possible to be conveniently elongated to other loss functions. Quite apparently, varying options of penalty *R*(Θ) are likely to result in significantly varying multitask methodologies. Based on some previous knowledge, we subsequently add penalty *R*(Θ) to encode the relatedness among tasks.

### 2.2. *G*_2,1_-Norm

One of the attractive properties of the *ℓ*_2,1_-norm regularization indicates that it provides multiple predictors from varying tasks with encouragement for sharing the same kind of parameter sparsity patterns. The *ℓ*_2,1_-norm regularization considers

(3) $$\begin{array}{c} {\left\|\Theta \right\|}_{2,1}=\overset{p}{\underset{j=1}{\sum }}{\left\|\theta_{j.}\right\|}_{2}, \end{array}$$

and is appropriate to concurrently enforce sparsity over the attributes of each task.

The primary point of equation (3) involves using *ℓ*_2_-norm for *θ*_*j*._, forcing the weights that correspond to the *j*-th attribute across multiple tasks for being grouped, besides being inclined to selecting the attributes based on the robustness of *k* tasks collectively. Besides, there is a relationship existing among multiple cognitive tests. As per a hypothesis, a pertinent imaging predictor usually more or less impacts each of these scores; furthermore, there is just a subset of brain regions having relevance to each evaluation. Through the use of the *ℓ*_2,1_-norm, the relationship information among varying tasks can be embedded into the framework to build a more suitable predictive framework, together with identifying a subset of the attributes. The rows of Θ receive equal treatment in *ℓ*_2,1_-norm, suggesting that the potential structures among predictors are not taken into consideration.

In spite of the achievements mentioned earlier, there are few regression frameworks, which consider the covariance structure among predictors. Aimed at attaining a specific feature, the brain imaging measures usually correlate with one another. Concerning the MRI data, the groups are respective to certain regions of interest (ROIs) in the brain, for instance, the entorhinal and hippocampus. Individual attributes are specific properties of those areas, for example, cortical volume as well as thickness. With regard to each area (group), multiple attributes are derived for the measurement of the atrophy information for all of the ROIs that involve cortical thickness, in addition to surface area and volume from gray matters as well as white matters in the current research work. The multiple shape measures from the same region provide a comprehensively quantitative evaluation of cortical atrophy and tend to be selected together as joint predictors [23].

We assume that *p* covariates are segregated into the *q* disjoint groups *𝒢*_*l*_,  *l*=1,…, *q* wherein every group has *ν*_*l*_ covariates, correspondingly. In the backdrop of AD, every group is respective to a region of interest (ROI) in the brain; furthermore, the covariates of all the groups are in respect to particular attributes of that area. Concerning AD, the number of attributes in every group, *ν*_*l*_, is 1 or 4, whereas the number of groups *q* is likely to be in hundreds. After that, we provide the introduction of two varying *G*_2,1_-norms in accordance with the correlation that exists between the brain regions (ROIs) and cognitive tasks: ‖Θ‖_*G*_2,1__^*c*^ encouraging a shared subset of ROIs for all the tasks and *ℓ*_2,1_ encouraging a task-specific subset of ROIs.

The task-common *G*_2,1_-norm ‖Θ‖_*G*_2,1__^*c*^ is defined as

(4) $$\begin{array}{c} {\left\|\Theta \right\|}_{G_{2,1}}^{c}=\overset{q}{\underset{l=1}{\sum }}w_{l}\sqrt{\underset{j\in G_{l}}{\sum }{\left\|\theta_{j.}\right\|}_{2}}, \end{array}$$

where $w_{l}=\sqrt{\nu_{l}}$ is the weight of each group. The task-common *G*_2,1_-norm enforces *ℓ*_2_-norm at the features within the same ROI (intragroup) and keeps sparsity among the ROIs (intergroup) with *ℓ*_1_ norm, to facilitate the selection of ROI. ‖Θ‖_*G*_2,1__^*c*^ allows to learn the shared feature representations as well as ROI representations simultaneously.

**Figure 2:**
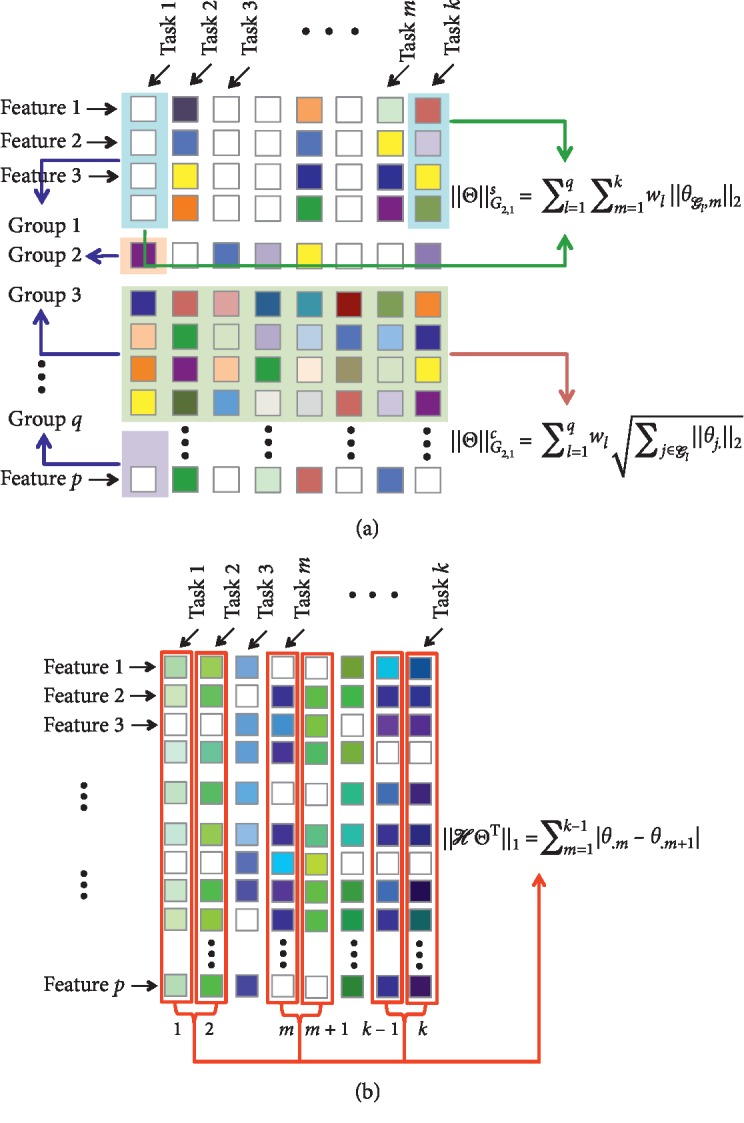
The illustration of three different regularizations. Each column of Θ is corresponding to a single task and each row represents a feature dimension. For each element in Θ, white color means zero-valued elements and color indicates nonzero values. (a) *G*_2,1_-norm. (b) Fused Lasso.

The task-specific *G*_2,1_-norm ‖Θ‖_*G*_2,1__^*s*^ is defined as

(5) $$\begin{array}{c} {\left\|\Theta \right\|}_{G_{2,1}}^{s}=\overset{q}{\underset{l=1}{\sum }}\overset{k}{\underset{m=1}{\sum }}w_{l}{\left\|\theta_{G_{l}m}\right\|}_{2}, \end{array}$$

where *θ*_*𝒢*_*l*_,*m*_ ∈ *ℝ*^*ν*_*l*_^ is the coefficient vector for group *𝒢*_*l*_ and task *m*. The task-specific *G*_2,1_-norm allows to select specific ROIs while learning a small number of common features for all tasks. It has more flexibility, which decouples the group sparse regularization across tasks, so that different tasks can use different groups. The difference between these two norms is illustrated in Figure 2(a).

### 2.3. Fused Lasso

Fused Lasso was first proposed by Tibshirani et al. [25]. Fused Lasso is one of the variants, where pairwise differences between variables are penalized using the *ℓ*_1_ norm, which results in successive variables being similar. The fused Lasso norm is defined as

(6) $$\begin{array}{c} {\left\|ℋ\Theta^{T}\right\|}_{1}=\overset{k-1}{\underset{m=1}{\sum }}\left\|\theta_{.m}-\theta_{.m+1}\right\|, \end{array}$$

where *ℋ* is a (*k* − 1) × *k* sparse matrix with *ℋ*_*m*,*m*_=1, and *ℋ*_*m*,*m*+1_=−1. It encourages *θ*_.*m*_ and *θ*_.*m*+1_ to take the same value by shrinking the difference between them toward zero. This approach has been employed to incorporate temporal smoothness to model disease progression. In longitudinal model, it is assumed that the difference of the cognitive scores between two successive time points is relatively small. The fused Lasso norm is illustrated in Figure 2(b).

## 3. Group Guided Fused Laplacian Sparse Group Lasso (GFL-SGL)

### 3.1. Formulation

In longitudinal studies, the cognitive scores of the same subject are measured at several time points. Consider a multitask learning problem over *k* tasks, where each task corresponds to a time point *t*=1,…, *k*. For each time point *t*, we consider a regression task based on data (*X*_*t*_, *y*_*t*_), where *X*_*t*_ ∈ *ℝ*^*n*×*p*^ denotes the matrix of covariates and *y*_*t*_ ∈ *ℝ*^*n*^ is the matrix of responses. Let Θ ∈ *ℝ*^*p*×*k*^ denote the regression parameter matrix over all tasks so that column *θ*_.*t*_ ∈ *ℝ*^*p*^ corresponds to the parameters for the task in time step *t*. By considering the prediction of cognitive scores at a single time point as a regression task, tasks at different time points are temporally related to each other. To encode the dependency graphs among all the tasks, we construct the Laplacian fused regularized penalty:

(7) $$\begin{array}{c} {\left|\left|\Theta D\right|\right|}_{1}=\overset{k}{\underset{t=1}{\sum }}{\left\|\theta_{.t}-\overset{k}{\underset{\begin{array}{c} \ell =1\\ \ell \neq t \end{array}}{\sum }}w_{\ell ,t}\theta_{.\ell }\right\|}_{1}, \end{array}$$

where *𝒟* ∈ *ℝ*^*k*×*k*^ has the following form:

(8) $$\begin{array}{c} \left[\begin{array}{ccccc} 1 & -w_{1,2} & -w_{1,3} & \cdots & -w_{1,k}\\ -w_{2,1} & 1 & -w_{2,3} & \cdots & -w_{2,k}\\ \vdots & \vdots & \vdots & \vdots & \vdots \\ -w_{k,1} & -w_{k,2} & -w_{k,3} & \cdots & 1 \end{array}\right]. \end{array}$$

We assume a viewpoint that is under inspiration from the local nonparametric regression, being specific, the kernel-based linear smoothers like the Nadaraya–Watson kernel estimator [26]. Considering this kind of view, we model the local approximation as

(9) $$\begin{array}{c} w_{\ell ,t}=\frac{\exp\left(-\left({\left(\ell -t\right)}^{2}/\sigma^{2}\right)\right)}{\sum_{\underset{\ell^{\prime }\neq t}{\ell^{\prime }=1}}^{k}\exp\left(-\left({\left(\ell^{\prime }-t\right)}^{2}/\sigma^{2}\right)\right)},\;\ell ,\;\ell^{\prime }=1,\ldots ,k,\;\ell \neq t. \end{array}$$

In our current work, weights are figured out with the help of a Gaussian kernel, as stated in equation (9), wherein *σ* indicates the kernel bandwidth, which requires a mandatory definition. As *σ* is small, the Gaussian curve shows a quick decay, followed by subsequent rapid decline of the weights *w*_|*t* − *ℓ*|_ with the increasing |*t* − *ℓ*|; conversely, as *σ* is large, the Gaussian curve shows a gradual decay, followed by the subsequent slow decline of the weights *w*_|*t* − *ℓ*|_ with the increasing |*t* − *ℓ*|. In this manner, the matrix *𝒟* shares symmetry with *w*_*t*,*ℓ*_=*w*_*ℓ*,*t*_, as an attribute of |*t* − *ℓ*|. Taking into account the covariance structure among predictors, we extend the Laplacian fused norm into group guided Laplacian fused norm.

The task-specific *G*_2,1_-norm was used here to decouple the group sparse regularization across tasks. *G*_2,1_-norm allows for more flexibility so that different fused tasks are regularized by different groups. The group guided fused Laplacian (GFL) regularization is defined as

(10) $$\begin{array}{c} {\left|\left|\Theta D\right|\right|}_{G_{2,1}^{F}}=\overset{k}{\underset{t=1}{\sum }}\overset{q}{\underset{l=1}{\sum }}w_{l}{\left\|\theta_{G_{l}t}-\overset{k}{\underset{\begin{array}{c} \ell =1\\ \ell \neq t \end{array}}{\sum }}w_{\ell ,t}\theta_{G_{l}\ell }\right\|}_{2}. \end{array}$$

**Figure 3:**
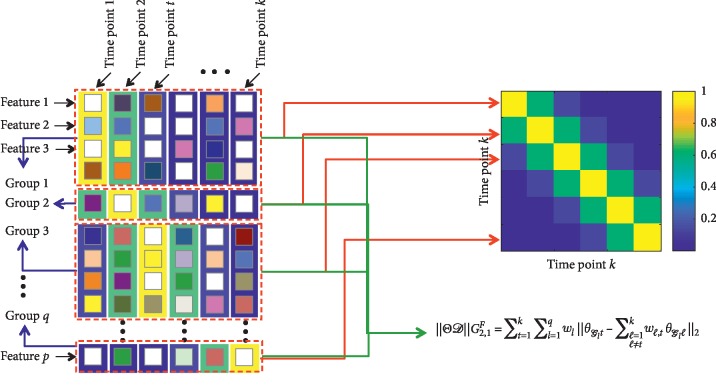
The illustration of GFL regularization. The regularization involves two matrices: parameter matrix (left); Gaussian kernel weighted fused Laplacian matrix with *σ*=1 (right).

The GFL regularization enforces *ℓ*_2_-norm at the fused features within the same ROI and keeps sparsity among the ROIs with *ℓ*_1_-norm to facilitate the selection of ROI. The GFL regularization is illustrated in Figure 3. The regularization involves two matrices: (1) Parameter matrix (left). For convenience, we let each group correspond to a time point in the transformation matrix. In fact, the transformation matrix operates on all groups. (2) Gaussian kernel weighted fused Laplacian matrix with *σ*=1 (right). Since this matrix is symmetric, we represent the columns as rows.

The clinical score data are incomplete at some time points for many patients, i.e., there may be no values in the target vector *y*_*i*_ ∈ *ℝ*^*k*^. In order not to reduce the number of samples significantly, we use a matrix Λ ∈ *ℝ*^*n*×*k*^ to indicate incomplete target vector instead of simply removing all the patients with missing values. Let Λ_*i*,*j*_=0 if the target value of sample *i* is missing at the *j*-th time point, and Λ_*i*,*j*_=1 otherwise. We use the componentwise operator  ⊙  as follows: *Z*=*A* ⊙ *B* denotes *z*_*i*,*j*_=*a*_*i*,*j*_*b*_*i*,*j*_, for all *i*, *j*. Then, plugging task-common *G*_2,1_-norm Θ_*G*_2,1__^*c*^ and Lasso to GFL model, the objective function of group guided fused Laplacian sparse group Lasso (GFL-SGL) is given in the following optimization problem:

(11) $$\begin{array}{c} \underset{\Theta }{\min}\frac{1}{2}{\left\|\Lambda \;⊙\;\left(Y-X\Theta \right)\right\|}_{F}^{2}+R_{\lambda_{2}}^{\lambda_{1}}\left(\Theta \right)+\lambda_{3}{\left\|\Theta D\right\|}_{G_{2,1}^{F}}, \end{array}$$

where *R*_*λ*_2__^*λ*_1_^(Θ)=*λ*_1_‖Θ‖_1_+*λ*_2_‖Θ‖_*G*_2,1__^*c*^ and *λ*_1_, *λ*_2_, *λ*_3_ are the regularization parameters.

### 3.2. Efficient Optimization for GFL-SGL

#### 3.2.1. ADMM

Recently, ADMM has emerged as quite famous since parallelizing the distributed convex issues is quite convenient usually. Concerning ADMM, the solutions to small local subproblems are coordinated to identify the global best solution [27–29]:

(12) $$\begin{array}{c} \begin{array}{cc} \underset{x,z}{\min} & f\left(x\right)+g\left(z\right),\\ \text{s.t.} & Ax+Bz=c. \end{array} \end{array}$$

The formulation of the variant augmented Lagrangian of ADMM methodology is done as follows:

(13) $$\begin{array}{c} L_{\rho }\left(x,z,u\right)=f\left(x\right)+g\left(z\right)+u^{T}\left(Ax+Bz-c\right)+\frac{\rho }{2}{\left\|Ax+Bz-c\right\|}^{2}, \end{array}$$

where *f* and *g* indicate the convex attributes and variables *A* ∈ *ℝ*^*p*×*n*^, *x* ∈ *ℝ*^*n*^, *B* ∈ *ℝ*^*p*×*m*^, *z* ∈ *ℝ*^*m*^, *c* ∈ *ℝ*^*p*^. *u* denotes a scaled dual augmented Lagrangian multiplier, whereas *ρ* suggests a nonnegative penalty parameter. In all of the iterations of ADMM, this issue is solved through the alternation of minimization *L*_*ρ*_(*x*, *z*, *u*) over *x*,  *z*,  and *u*. Concerning the (*k*+1)-th iteration, ADMM is updated by

(14) $$\begin{array}{c} x^{k+1}≔\underset{x}{\arg\;\min}L_{\rho }\left(x,z^{k},u^{k}\right),\\ z^{k+1}≔\underset{z}{\arg\;\min}L_{\rho }\left(x^{k+1},z,u^{k}\right),\\ u^{k+1}≔u^{k}+\rho \left(Ax^{k+1}+Bz^{k+1}-c\right). \end{array}$$

#### 3.2.2. Efficient Optimization for GFL-SGL

We put forward an efficient algorithm to solve the objective function in equation (11), equaling the limited optimization issue as follows:

(15) $$\begin{array}{c} \begin{array}{cc} \underset{\Theta ,Q,\Gamma }{\min} & \frac{1}{2}{\left\|\Lambda \;⊙\;\left(Y-X\Theta \right)\right\|}_{F}^{2}+R_{\lambda_{2}}^{\lambda_{1}}\left(Q\right)+\lambda_{3}{\left\|\Gamma \right\|}_{G_{2,1}^{F}},\\ \text{s.t.} & \begin{array}{c} \Theta -Q=0,\\ \Theta D-\Gamma =0, \end{array} \end{array} \end{array}$$

where *Q*,  Γ refer to slack variables. After that, the solution of equation (15) can be obtained by ADMM. The augmented Lagrangian is

(16) $$\begin{array}{c} {ℒ}_{\rho }\left(\Theta ,Q,\Gamma ,U,V\right)=\frac{1}{2}{\left\|\Lambda \;⊙\;\left(Y-X\Theta \right)\right\|}_{F}^{2}+R_{\lambda_{2}}^{\lambda_{1}}\left(Q\right)+\lambda_{3}{\left\|\Gamma \right\|}_{G_{2,1}^{F}}+\langle U,\Theta -Q\rangle +\frac{\rho }{2}{\left\|\Theta -Q\right\|}^{2}+\langle V,\Theta D-\Gamma \rangle +\frac{\rho }{2}{\left\|\Theta D-\Gamma \right\|}^{2}, \end{array}$$

where *U*,  *V* are augmented Lagrangian multipliers.

*Update*Θ: from the augmented Lagrangian in equation (16), the update of Θ at (*s*+1)-th iteration is conducted by

(17) $$\begin{array}{c} \Theta^{\left(s+1\right)}=\arg\;\underset{\Theta }{\min}\frac{1}{2}{\left\|\Lambda \;⊙\;\left(Y-X\Theta \right)\right\|}_{F}^{2}+\langle U^{\left(s\right)},\Theta -Q^{\left(s\right)}\rangle +\frac{\rho }{2}{\left\|\Theta -Q^{\left(s\right)}\right\|}^{2}+\langle V^{\left(s\right)},\Theta D-\Gamma^{\left(s\right)}\rangle +\frac{\rho }{2}{\left\|\Theta D-\Gamma^{\left(s\right)}\right\|}^{2}, \end{array}$$

that is a closed form, which is likely to be extracted through the setting of equation (17) to zero.

(18) $$\begin{array}{c} 0=-\Lambda \;⊙\;X^{T}\left(Y-X\Theta \right)+U^{\left(s\right)}+\rho \left(\Theta -Q^{\left(s\right)}\right)+V^{\left(s\right)}D+\rho \left(\Theta D-\Gamma^{\left(s\right)}\right)D. \end{array}$$

It requires observation that *𝒟* indicates a symmetric matrix. Besides, we state Φ=*𝒟𝒟*, wherein Φ is also an indication of a symmetric matrix where Φ_*t*,*l*_ denotes the value of weight (*t*, *l*). Through this kind of a linearization, Θ can be updated in parallel with the help of the individual *θ*_.*t*_. In this manner, in the (*s*+1)-th iteration, it is possible to update *θ*_.*t*_^(*s*+1)^efficiently with the use of Cholesky.

(19) $$\begin{array}{c} 0=-X^{T}\left(y_{t}-X\theta_{.t}\right)+u_{.t}^{\left(s\right)}+\rho \left(\theta_{.t}-q_{.t}^{\left(s\right)}\right)+\left(v_{.t}^{\left(s\right)}-\overset{k}{\underset{\begin{array}{c} t=1\\ t\neq l \end{array}}{\sum }}D_{t,l}v_{.l}^{\left(s\right)}\right)+\rho \left(\Phi_{t,t}\theta_{.t}-\overset{k}{\underset{\begin{array}{c} t=1\\ t\neq l \end{array}}{\sum }}\Phi_{t,l}\theta_{.l}\right)-\rho \left(\gamma_{.t}^{\left(s\right)}-\overset{k}{\underset{\begin{array}{c} t=1\\ t\neq l \end{array}}{\sum }}D_{t,l}\gamma_{.l}^{\left(s\right)}\right). \end{array}$$

The above optimization problem is quadratic. The optimal solution is given by *θ*_.*t*_^(*s*+1)^=*F*_*t*_^−1^*b*_*t*_^(*s*)^, where

(20) $$\begin{array}{c} F_{t}=X^{T}X+\rho \left(1+\Phi_{t,t}\right)I,\\ b_{t}^{\left(s\right)}=X^{T}y_{t}-u_{.t}^{\left(s\right)}-\left(v_{.t}^{\left(s\right)}-\overset{k}{\underset{\begin{array}{c} t=1\\ t\neq l \end{array}}{\sum }}D_{t,l}v_{.l}^{\left(s\right)}\right)+\rho q_{t}^{\left(s\right)}+\rho \left(\gamma_{.t}^{\left(s\right)}-\overset{k}{\underset{\begin{array}{c} t=1\\ t\neq l \end{array}}{\sum }}D_{t,l}\gamma_{.l}^{\left(s\right)}\right)+\rho \overset{k}{\underset{\begin{array}{c} t=1\\ t\neq l \end{array}}{\sum }}\Phi_{t,l}\theta_{.l}. \end{array}$$

Computing *θ*_.*t*_^(*s*+1)^ deals with the solution of a linear system, the most time-consuming component in the entire algorithm. For the computation of *θ*_.*t*_^(*s*+1)^ in an efficient manner, we perform the calculation of the Cholesky factorization of *F* as the algorithm begins:

(21) $$\begin{array}{c} F_{t}=A_{t}^{T}A_{t}. \end{array}$$

Observably, *F* refers to a constant and positive definite matrix. With the use of the Cholesky factorization, we require solving the following two linear systems at all of the iterations:

(22) $$\begin{array}{c} A_{t}^{T}{\hat{\theta }}_{.t}=b^{\left(s\right)},\\ A\theta_{.t}={\hat{\theta }}_{.t}. \end{array}$$

Accordingly, *A*_*t*_ indicates an upper triangular matrix, which solves these two linear systems, which is quite effective.

*Update Q*: updating *Q* effectively requires solving the problem as follows:

(23) $$\begin{array}{c} Q^{\left(s+1\right)}=\text{arg}\underset{Q}{\text{min}}\frac{\rho }{2}{\left\|Q-\Theta^{\left(s+1\right)}\right\|}^{2}+R_{\lambda_{2}}^{\lambda_{1}}\left(Q\right)-\langle U^{\left(s\right)},Q\rangle , \end{array}$$

which equals the computation of the proximal operator for *R*_*λ*_2__^*λ*_1_^(·). Being specific, we require solving

(24) $$\begin{array}{c} \Psi_{\lambda_{2}/\rho }^{\lambda_{1}/\rho }\left(\Omega^{\left(s+1\right)}\right)=\arg\underset{Q}{\min}\left\{R_{\lambda_{2}/\rho }^{\lambda_{1}/\rho }\left(Q\right)+\frac{1}{2}{\left\|Q-\Omega^{\left(s+1\right)}\right\|}^{2}\right\}, \end{array}$$

where *Ω*^(*s*+1)^=Θ^(*s*+1)^+(1/*ρ*)*U*^(*s*)^. This is aimed at being capable of computing *Q*^(*s*+1)^=Ψ_*λ*_2_/*ρ*_^*λ*_1_/*ρ*^(*Ω*^(*s*+1)^) in an efficient manner. The computation of the proximal operator for the composite regularizer can be done effectively in two steps [30, 31], which are illustrated as follows:

(25a) $$\begin{array}{c} \Pi^{\left(s+1\right)}=\Psi_{0}^{\lambda_{1}/\rho }\left(\Omega^{\left(s+1\right)}\right), \end{array}$$

(25b) $$\begin{array}{c} Q^{\left(s+1\right)}=\Psi_{\lambda_{2}/\rho }^{0}\left(\Pi^{\left(s+1\right)}\right)=\Psi_{\lambda_{2}/\rho }^{\lambda_{1}/\rho }\left(\Omega^{\left(s+1\right)}\right). \end{array}$$

These two steps can be carried out efficiently with the use of suitable extensions of soft-thresholding. It is possible to compute the update in equation (25a) with the help of the soft-thresholding operator *ζ*_*λ*_1_/*ρ*_(*Ω*^(*s*+1)^), which is stated as follows:

(26) $$\begin{array}{c} \zeta_{\lambda }\left(x\right)=\text{sign}\left(x\right)\max\left(\left|x\right|-\lambda ,\;0\right). \end{array}$$

After that, we emphasize updating equation (25b), effectively equivalent to the computation of the proximal operator for *G*_2,1_-norm. Specifically, the problem can be jotted down as follows:

(27) $$\begin{array}{c} Q^{\left(s+1\right)}=\underset{Q}{\arg\;\min}\left\{\frac{\lambda_{2}}{\rho }{\left\|Q\right\|}_{G_{2,1}}^{c}+\frac{1}{2}\left\|Q-\Pi^{\left(s+1\right)}\right\|\right\}. \end{array}$$

Since group *𝒢*_*ℓ*_ put to use in our research work is disjoint, equation (27) can be decoupled into

(28) $$\begin{array}{c} q_{j.}^{\left(s+1\right)}=\text{arg }\underset{q_{j.}}{\text{min}}\phi \left(q_{j.}\right)=\text{arg }\underset{q_{j.}}{\text{min}}\left\{\frac{1}{2}{\left\|q_{j.}-\pi_{j.}^{\left(s+1\right)}\right\|}^{2}+\frac{\lambda_{2}}{\rho }\left\|q_{j.}\right\|\right\}. \end{array}$$

Because *ϕ*(*q*_*j*._) is strictly convex, we conclude that *q*_*j*._^(*s*+1)^ refers to its exclusive minimizer. After that, we provide the introduction of the following lemma [32] for the solution of equation (28).

**Lemma 1 .**

For any *λ*_2_ ≥ 0, we have

(29) $$\begin{array}{c} q_{j.}=\frac{\max\left\{\sqrt{\sum_{j\in G_{l}}{\left\|\pi_{j.}\right\|}_{2}^{2}}-\left(\lambda_{2}w_{l}/\rho \right),0\right\}}{\sqrt{\sum_{j\in G_{l}}{\left\|\pi_{j.}\right\|}_{2}^{2}}}\pi_{j.}, \end{array}$$

where *q*_*j*._ is the *j*-th row of *Q*^*s*+1^.

*Update*Γ: the update for Γ efficiently requires solving the problem as follows:

(30) $$\begin{array}{c} \Gamma^{\left(s+1\right)}=\arg\;\underset{\Gamma }{\min}\frac{\rho }{2}{\left\|\Theta^{\left(s+1\right)}D-\Gamma \right\|}^{2}+\lambda_{3}{\left\|\Gamma \right\|}_{G_{2,1}^{F}}-\langle V^{\left(s\right)},\Gamma \rangle , \end{array}$$

which is efficiently equivalent to the computation of the proximal operator for GFL-norm. Explicitly, the problem can be stated as follows:

(31) $$\begin{array}{c} \Gamma^{\left(s+1\right)}=\arg\;\underset{\Gamma }{\min}\left\{\frac{\lambda_{3}}{\rho }{\left\|\Gamma \right\|}_{G_{2,1}^{F}}+\frac{1}{2}\left\|\Gamma -Z\right\|\right\}, \end{array}$$

where *Z*^(*s*+1)^=Θ^(*s*+1)^*𝒟*+(1/*ρ*)*V*^(*s*)^. Equation (31) can be decoupled into

(32) $$\begin{array}{c} \gamma_{G_{l}t}^{\left(s+1\right)}=\arg\;\underset{\gamma_{G_{l}t}}{\min}\phi \left(\gamma_{G_{l}t}\right)=\arg\;\underset{\gamma_{G_{l}t}}{\min}\left\{\frac{1}{2}{\left\|\gamma_{G_{l}t}-z_{G_{l}t}^{\left(s+1\right)}\right\|}^{2}+\frac{\lambda_{3}}{\rho }\left\|\gamma_{G_{l}t}\right\|\right\}. \end{array}$$

Then, we introduce the following lemma [32].

**Lemma 2 .**

For any *λ*_3_ ≥ 0, we have

(33) $$\begin{array}{c} \gamma_{G_{\ell }t}=\frac{\text{max}\left\{{\left\|z_{G_{\ell }t}\right\|}_{2}-\left(\lambda_{3}w_{\ell }/\rho \right),0\right\}}{{\left\|z_{G_{\ell }t}\right\|}_{2}}z_{G_{\ell }t}, \end{array}$$

where *γ*_*𝒢*_*ℓ*_*t*_, *z*_*𝒢*_*ℓ*_*t*_ are rows in group *𝒢*_*ℓ*_ for task *t* of Γ^(*s*+1)^ and *Z*^(*s*+1)^, respectively.

Dual update for *U* and *V*: following the standard ADMM dual update, the update for the dual variable for our setting is presented as follows:

(34a) $$\begin{array}{c} U^{\left(s+1\right)}=U^{\left(s\right)}+\rho \left(\Theta^{\left(s+1\right)}-Q^{\left(s+1\right)}\right), \end{array}$$

(34b) $$\begin{array}{c} V^{\left(s+1\right)}=V^{\left(s\right)}+\rho \left(\Theta^{\left(s+1\right)}D-\Gamma^{\left(s+1\right)}\right). \end{array}$$

It is possible to carry out the dual updates in an elementwise parallel way.  Algorithm 1 provides a summary of the entire algorithm. MATLAB codes of the proposed algorithm are available at https://XIAOLILIU@bitbucket.org/XIAOLILIU/gfl-sgl.

### 3.3. Convergence

The convergence of the Algorithm 1 is shown in the following lemma.

**Theorem 3 .**

*Suppose there exists at least one solution *Θ^*∗*^* of equation (11). Assume 𝒢*_*ℓ*_*is convex*, *λ*_1_ > 0, *λ*_2_ > 0, *λ*_3_ > 0.*Then the following property for GFL-SGL iteration in Algorithm 1 holds:*

(35) $$\begin{array}{c} \underset{s⟶\infty }{\lim}L\left(\Theta^{\left(s\right)}\right)+R_{\lambda_{2}}^{\lambda_{1}}\left(\Theta^{\left(s\right)}\right)+\lambda_{3}{\left\|\Theta^{\left(s\right)}D\right\|}_{G_{2,1}^{F}}\\ =L\left(\Theta^{*}\right)+R_{\lambda_{2}}^{\lambda_{1}}\left(\Theta^{*}\right)+\lambda_{3}{\left\|\Theta^{*}D\right\|}_{G_{2,1}^{F}}. \end{array}$$

Furthermore,

(36) $$\begin{array}{c} \underset{s⟶\infty }{\lim}\left\|\Theta^{\left(s\right)}-\Theta^{*}\right\|=0, \end{array}$$

whenever equation (11) has a unique solution.

The condition allowing the convergence in Theorem 1 is very convenient to meet. *λ*_1_,  *λ*_2_, and *λ*_3_ refer to the regularization parameters, which are required to be above zero all the time. The detailed proof is elaborated in Cai et al. [33]. Contrary to Cai et al., we do not need *L*(Θ) as differentiable, in addition to explicitly treating the nondifferentiability of *L*(Θ) through the use of its subgradient vector ∂*L*(Θ), which shares similarity with the strategy put to use by Ye and Xie [28].

## 4. Experimental Results and Discussions

In this section, we put forward the empirical analysis for the demonstration of the efficiency of the suggested model dealing with the characterization of AD progression with the help of a dataset from the Alzheimer's Disease Neuroimaging Initiative (ADNI) [34]. The principal objective of ADNI has been coping with testing if it is possible to combine serial MRI, together with PET, other biological markers, and medical and neuropsychological evaluations to measure the progression of MCI as well as early AD. Approaches for the characterization of the AD progression are expected to assisting both researchers and clinicians in developing new therapies and monitoring their efficacies. Besides, being capable of understanding the disease progression is expected to augment both the safety and efficiency of the drug development, together with potentially lowering the time and cost associated with the medical experiments.

### 4.1. Experimental Setup

The ADNI project is termed as a longitudinal research work, in which the chosen subjects are classified into three baseline diagnostic cohorts that include Cognitively Normal (CN), Mild Cognitive Impairment (MCI), and Alzheimer's Disease (AD), recurrently encompassing the interval of six or twelve months. Also, the date of scheduling the subjects for performing the screening emerges as the baseline (BL) after that approval; also, the time point for the follow-up visits is indicated by the period time that starts from the baseline. Moreover, we put to use the notation Month 6 (M6) to denote the time point half year following the very first visit. Nowadays, ADNI possesses up to Month 48 follow-up data that some patients can avail. Nevertheless, some patients skip research work for several causes.

The current work places emphasis on the MRI data. Furthermore, the MRI attributes put to use in our assays are made based on the imaging data from the ADNI database that is processed with the help of a team from UCSF (University of California at San Francisco), carrying out cortical reconstruction as well as volumetric segmentations using the FreeSurfer image analysis suite (http://surfer.nmr.mgh.harvard.edu/). In the current investigation, we eliminate the attributes that have over 10% missing entries (concerning every patient as well as every time point), besides excluding the patients, who do not have the baseline MRI records and completing the missing entries with the use of the average value. This yields a total of *n*=788 subjects (173 AD, 390 MCI, and 225 CN) for baseline, and for the M6, M12, M24, M36, and M48 time points, the sample size is 718 (155 AD, 352 MCI, and 211 CN), 662 (134 AD, 330 MCI, and 198 CN), 532 (101 AD, 254 MCI, and 177 CN), 345 (1 AD, 189 MCI, and 155 CN), and 91 (0 AD, 42 MCI, and 49 CN), respectively. In aggregate, forty-eight cortical regions together with forty-four subcortical regions are created after this preprocessing. Both Tables 1 and 2 [19, 21] shed light on the names of the cortical and subcortical regions. For each cortical region, the cortical thickness average (TA), standard deviation of thickness (TS), surface area (SA), and cortical volume (CV) were calculated as features. For each subcortical region, subcortical volume was calculated as features. The SA of left and right hemisphere and total intracranial volume (ICV) were also included. This yielded a total of *p*=319 MRI features extracted from cortical/subcortical ROIs in each hemisphere (including 275 cortical and 44 subcortical features). Details of the analysis procedure are available at http://adni.loni.ucla.edu/research/mri-post-processing/.

For predictive modeling, five sets of cognitive scores [25, 35] are examined: Alzheimer's Disease Assessment Scale (ADAS), Mini-Mental State Exam (MMSE), Rey Auditory Verbal Learning Test (RAVLT), Category Fluency (FLU), and Trail Making Test (TRAILS). ADAS is termed as the gold standard in the AD drug experiment concerning the cognitive function evaluation that refers to the most famous cognitive testing tool for the measurement of the seriousness of the most pivotal signs of AD. Furthermore, MMSE measures cognitive damage, which includes orientation to both time and place, coupled with the attention and calculation, spontaneous and delayed recall of words, and language and visuoconstructional attributes. RAVLT refers to the measurement of the episodic memory and put to use to diagnose memory interruptions, comprising eight recall experiments as well as a recognition test. FLU refers to the measurement of semantic memory (verbal fluency and language). The subject is requested for naming varying exemplars from a provided semantic classification. Furthermore, TRAILS is termed as an array of processing speed and executive attribute, comprising two components, wherein the subject is directed for connecting a set of twenty-five dots at the fastest possible, meanwhile performing the maintenance of precision. The specific scores we used are listed in Table 3. Note that the proposed GFL-SGL models are trained to model progression for each of these scores, with different time steps serving the role of distinct tasks. Since the five sets of cognitive scores include a total of ten different scores (see Table 3), results will be reported on each of these ten scores separately.

Concerning all of the trials, 10-fold cross valuation is employed for the evaluation of our framework, together with carrying out the comparison. For all of the experiments, 5-fold cross validation on the training set is carried out to select the regularization parameters (hyperparameters) (*λ*_1_, *λ*_2_, *λ*_3_). The approximated framework makes use of these regularization parameters for the prediction on the experiment set. About the cross validation, concerning a fixed set of hyperparameters, the use of four folds is made to train, besides using one fold for assessment with the help of nMSE. Concerning the hyperparameter choice, we take into account a grid of regularization parameter values, in which every regularization parameter varies between 10^−1^ and 10^3^ in log scale. The data were z-scored before the application of the regression methods. The reported findings constituted the optimal findings of every method having the best parameter. Regarding the quantitative efficiency assessment, we made use of the metrics of correlation coefficient (CC) as well as root mean squared error (rMSE) between the forecasted medical scores and the targeted medical scores for all of the regression tasks. Besides, for the evaluation of the overall efficiency on each task, the use of normalized mean squared error (nMSE) [12, 24] and weighted R-value (wR) [36] is made. The nMSE and wR are defined as follows:

(37) $$\begin{array}{c} n\text{MSE}\left(Y,\hat{Y}\right)=\frac{\sum_{h=1}^{k}\left(\left({\left\|Y_{h}-{\hat{Y}}_{h}\right\|}_{2}^{2}\right)/\left(\sigma \left(Y_{h}\right)\right)\right)}{\sum_{h=1}^{k}n_{h}},\\ wR\left(Y,\hat{Y}\right)=\frac{\sum_{h=1}^{k}\text{Corr}\left(Y_{h},{\hat{Y}}_{h}\right)n_{h}}{\sum_{h=1}^{k}n_{h}}, \end{array}$$

where *Y* and $\hat{Y}$ are the ground truth cognitive scores and the predicted cognitive scores, respectively. A smaller (higher) value of nMSE and rMSE (CC and wR) represents better regression performance. We report the mean and standard deviation based on 10 iterations of experiments on different splits of data for all comparable experiments. We also performed paired *t*-tests on the corresponding cross validation performances measured by the nMSE and wR between predicted and actual scores to compare the proposed method and the other comparison methods [9, 24, 35, 37]. The *p* values were provided to examine whether these improved prediction performances were significant. A significant performance has a low *p* value (less than 0.05 for example).

**Figure 4:**
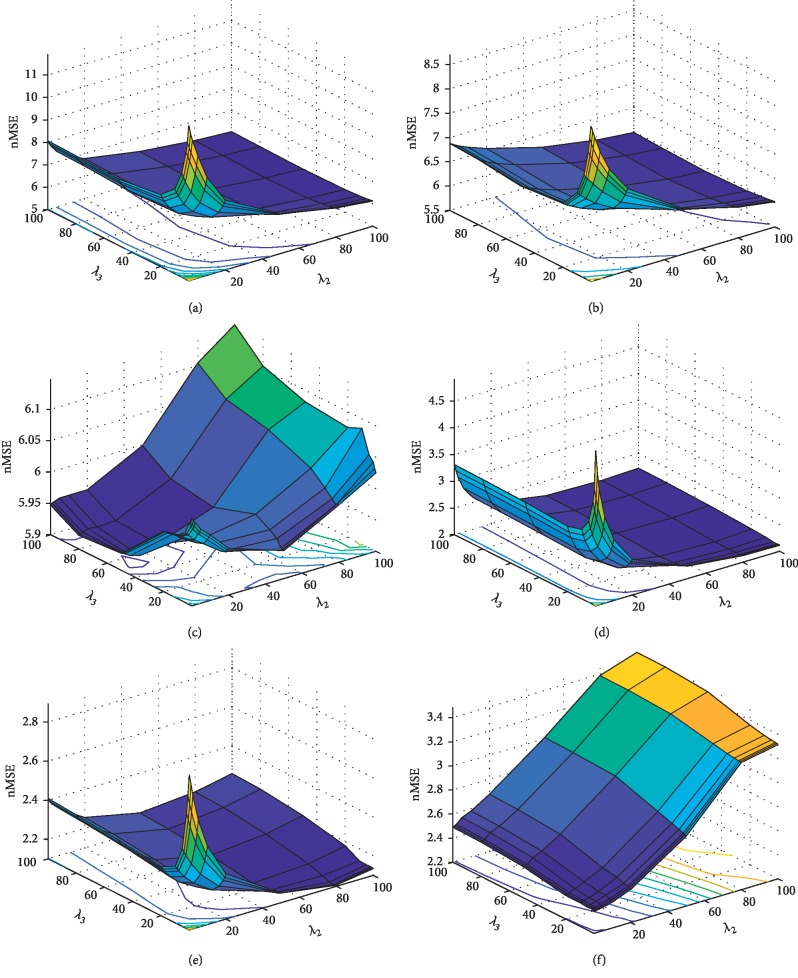
Hyperparameter sensitivity analysis: hyperparameter *λ*_3_ associated with the GFL-SGL temporal smooth penalization term mainly contributes to the forecast and requires consideration. Bigger values for *λ*_2_ (linked to the task-common group Lasso penalty) tends to enhance the findings for smaller *λ*_1_. (a) ADAS (*λ*_1_=1). (b) ADAS (*λ*_1_=10). (c) ADAS (*λ*_1_=100). (d) MMSE (*λ*_1_=1). (e) MMSE (*λ*_1_=10). (f) MMSE (*λ*_1_=100).

Aimed at assessing the sensitivity of the three hyperparameters in the GFL-SGL formulation (equation (11)), we investigated the 3D hyperparameter space, in addition to plotting the nMSE metric for all of the mixes of values, in the way we had done in our recent investigation [19]. The sensitivity research work is of importance for the study of the impact of all the terms in the GFL-SGL formulation, together with guiding on the way of appropriately setting the hyperparameters. The definition of the hyperparameter space is made as *λ*_1_, *λ*_2_, *λ*_3_ ∈ [0.1, 100]. The nMSE put forward was calculated in the test set. Owing to the space constraints, Figure 4 merely sheds light on the plots for ADAS as well as MMSE cognitive scores. Observing the fact is possible that, concerning all of the cognitive scores, smaller values for *λ*_3_ resulted in the low regression efficiency, which suggested that the temporal smooth penalization term mainly contributes to the forecast and requires consideration. Moreover, the bigger values for *λ*_2_ (linked to the task-common group Lasso penalty) tends to enhance the findings for smaller *λ*_1_. With the rise in *λ*_1_, we bring into force more sparsity on *θ* parameters, accordingly breaking the group structure that prevails in the data.

### 4.2. Prediction Performance Based on MRI Features

We compare the performance of GFL-SGL with different regression methods, including ridge regression [38] and Lasso [39], which are applied independently to each time point, and temporal group Lasso (TGL) [9] and convex fused sparse group Lasso (cFSGL) [24], which are state-of-the-art methods for characterizing longitudinal AD progression. TGL incorporates three penalty terms to capture task relatedness, which contains two *ℓ*_2_-norms to prevent overfitting and enforce temporal smoothness, and one *ℓ*_2,1_-norm to introduce joint feature selection. The optimal function is formulated as min_Θ_ *L*(Θ)+*λ*_1_‖Θ‖_*F*_^2^+*λ*_2_||*ℛ*Θ^*T*^||_*F*_^2^+*λ*_3_‖Θ‖_2,1_. cFSGL allows the simultaneous selection of a common set of biomarkers for multiple time points and specific sets of biomarkers for different time points using the sparse group Lasso (SGL, *λ*_1_||Θ||_2,1_+*λ*_2_‖Θ‖_1_) penalty and in the meantime incorporates the temporal smoothness using the fused Lasso penalty (∑_*t*=1_^*k*−1^|*θ*_*t*._ − *θ*_*t*+1._|). The downloading of the codes of TGL and cFSGL is carried out from the authors' websites, whereas the AGM algorithm is put to use as the optimization methodology. It is recalling the fact that every trial emphasizes a particular cognitive score, having varying time points that serve as different tasks for the multitask learning formulations. Since, in aggregate, there are ten cognitive scores, we carry out the trials, besides reporting the outcomes separately about all of the scores. The calculation of the average and standard deviation of the efficiency measures is carried out with the help of the 10-fold cross validation on the different splits of data, summarized in Table 4.

The results show that multitask temporal smoothness models (TGL, cFSGL, and GFL-SGL) are more effective than single-task learning models (ridge and Lasso) in terms of both nMSE and wR over all scores, especially for the task at the later time points where the training samples are limited. Both the norms of fused Lasso (TGL and cFSGL) and group guided fused Lasso (GFL-SGL) can improve performance, which demonstrates that taking into account the local structure within the tasks improves the prediction performance. Furthermore, GFL-SGL achieved better performances than TGL and cFSGL, which indicates that it is beneficial to simultaneously employ transform matrix taking into account all the time points and group structure information among the features. Two types of group penalties are used in our model (*G*_2,1_^*c*^-norm and *G*_2,1_^*F*^-norm). The former learns a shared subset of ROIs for all the tasks, whereas the latter learns a task-specific subset of Laplacian fused ROIs. Our GFL-SGL model performs consistently better than TGL and cFSGL, which further demonstrates that exploiting the underlying dependence structure may be advantageous, and exploiting the structure among tasks and features simultaneously resulted in significantly better prediction performance. The statistical hypothesis test reveals that GFL-SGL is significantly better than the contenders for most of the scores.

**Figure 5:**
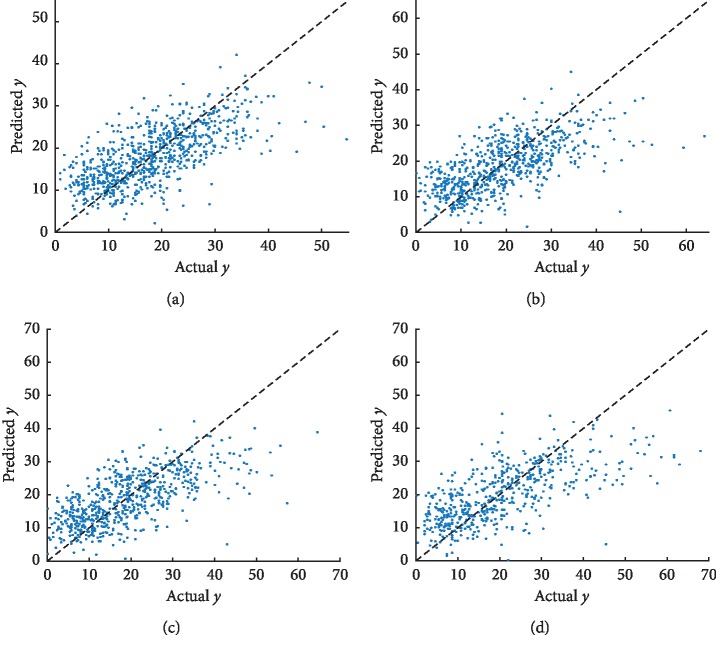
Scatter plots of the actual ADAS against the forecasted values on the test dataset by GFL-SGL using MRI features. High correlation is observed for the ADAS score at each time point. (a) Baseline (ADAS BL *R* = 0.678). (b) Month 6 (ADAS M6 *R* = 0.657). (c) Month 12 (ADAS M12 *R* = 0.673). (d) Month 24 (ADAS M24 *R* = 0.693).

**Figure 6:**
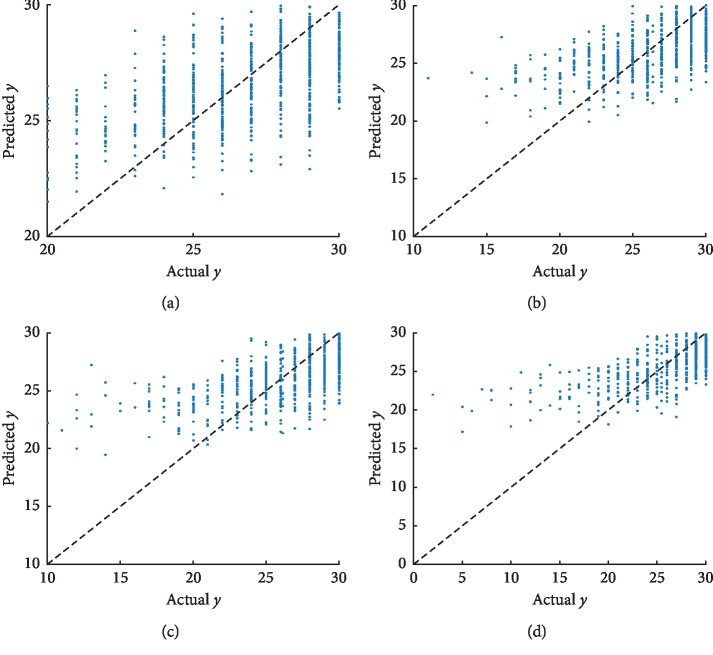
Scatter plots of the actual MMSE against the forecasted values on the test dataset by GFL-SGL using MRI features. High correlation is observed for the MMSE score at each time point. (a) Baseline (MMSE BL *R* = 0.574). (b) Month 6 (MMSE M6 *R* = 0.572). (c) Month 12 (MMSE M12 *R* = 0.609). (d) Month 24 (MMSE M24 *R* = 0.662).

We shed light on the scatter plots of the actual values against the forecasted values on the test dataset. For lacking the space, we just illustrated two scatter plots, which included ADAS as well as MMSE in Figures 5 and 6, correspondingly. Owing to the small sample size at M36 and M48 time points, we indicate the scatter plots for the first four time points. As the scatter plots indicate, the forecasted values, as well as the actual values scores, are similarly highly correlated to both of these tasks. The scatter plots demonstrate the fact that the prediction efficiency for ADAS is better as compared with that of MMSE.  Section 4.4 is going to incorporate more modalities, which include not just PET but also CSF and demographic information, aimed at improving efficiency.

### 4.3. Identification of MRI Biomarkers

**Figure 7:**
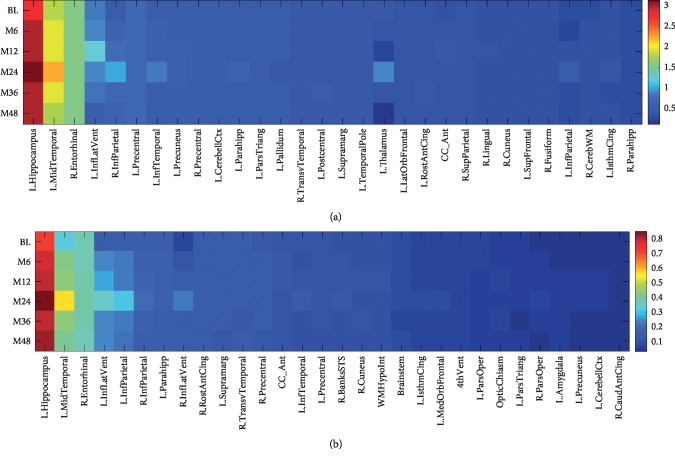
Longitudinal heat maps of regression coefficients generated by GFL-SGL for ADAS and MMSE using 10 trials on different splits of data. The larger the value is, the more important the ROI is. (a) ADAS. (b) MMSE.

In Alzheimer's disease research works, researchers have interest in the provision of the improved cognitive scores forecast, besides identifying which constitute the brain regions that are more impacted by the disease that has the potential of helping perform the diagnosis of the preliminary phases of the disease, besides its way of dissemination. After that, we revert to analyzing the identification of MRI biomarkers. Our GFL-SGL refers to a group sparse framework, capable of identifying a compact set of relevant neuroimaging biomarkers from the region level for the group Lasso on the attributes, which is expected to give us improved interpretability of the brain region. Due to lack of space, we only show the top 30 ROIs for ADAS and MMSE by obtaining the regression weights of all ROIs in each hemisphere for six time points in Figure 7. The value of each item (*i*, *j*) in the heat map indicates the weight of the *i*-th ROI for the *j*-th time point and is calculated by $w_{i}\sqrt{\sum_{k\in {𝒢}_{i}}{\left\|\theta_{ki}\right\|}_{2}}$, where *k* is the *k*-th MRI feature. The larger the absolute value of a coefficient is, the more important its corresponding brain region is in predicting the corresponding time point of that cognitive score. The figure illustrates that the proposed GFL-SGL clearly presents sparsity results across all time points, which demonstrates that these biomarkers are longitudinally important due to the advantage of smooth temporal regularization. We also observe that different time points share similar ROIs for these two cognitive measures, which demonstrates that there exists a strong correlation among the multiple tasks of score prediction at multiple time points.

**Figure 8:**
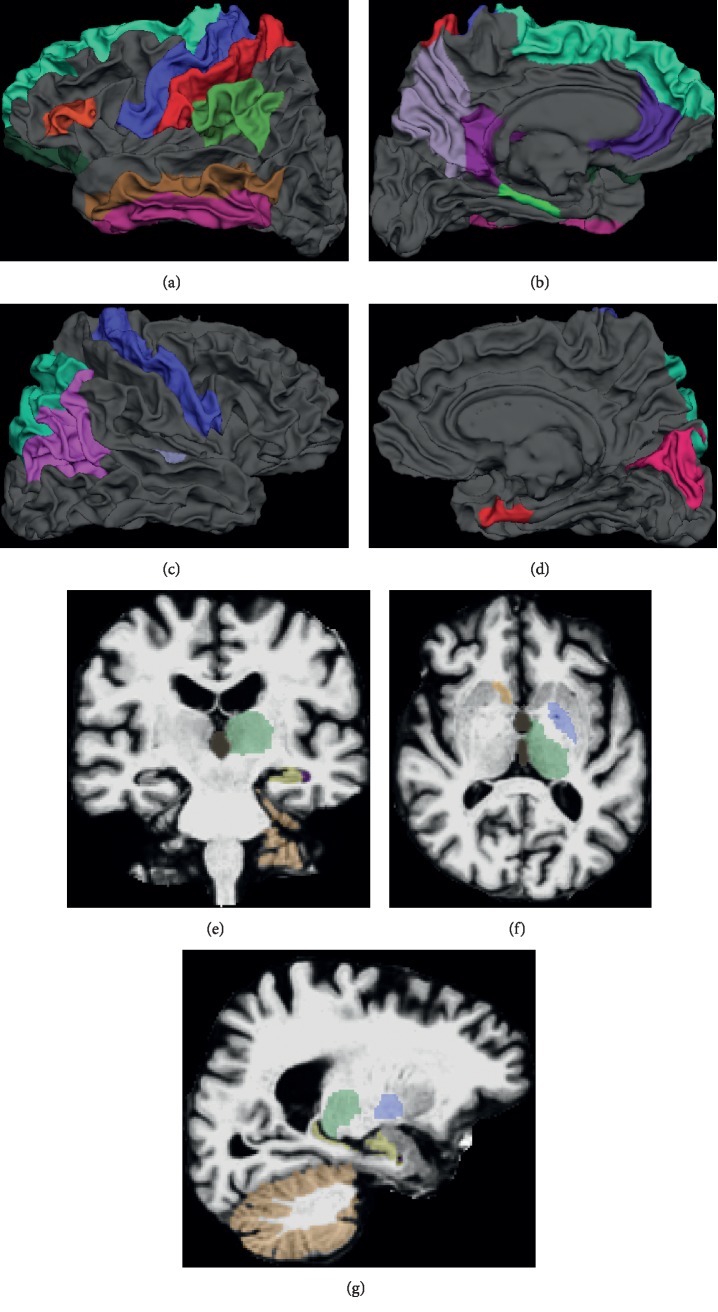
Brain maps of the top 30 ROIs selected by GFL-SGL for ADAS. (a)–(d) are cortical ROIs selected; (e)–(g) are subcortical ROIs selected. (a) Left hemisphere (outside). (b) Left hemisphere (inside). (c) Right hemisphere (outside). (d) Right hemisphere (inside). (e) Coronal view. (f) Horizontal view. (g) Sagittal view.

**Figure 9:**
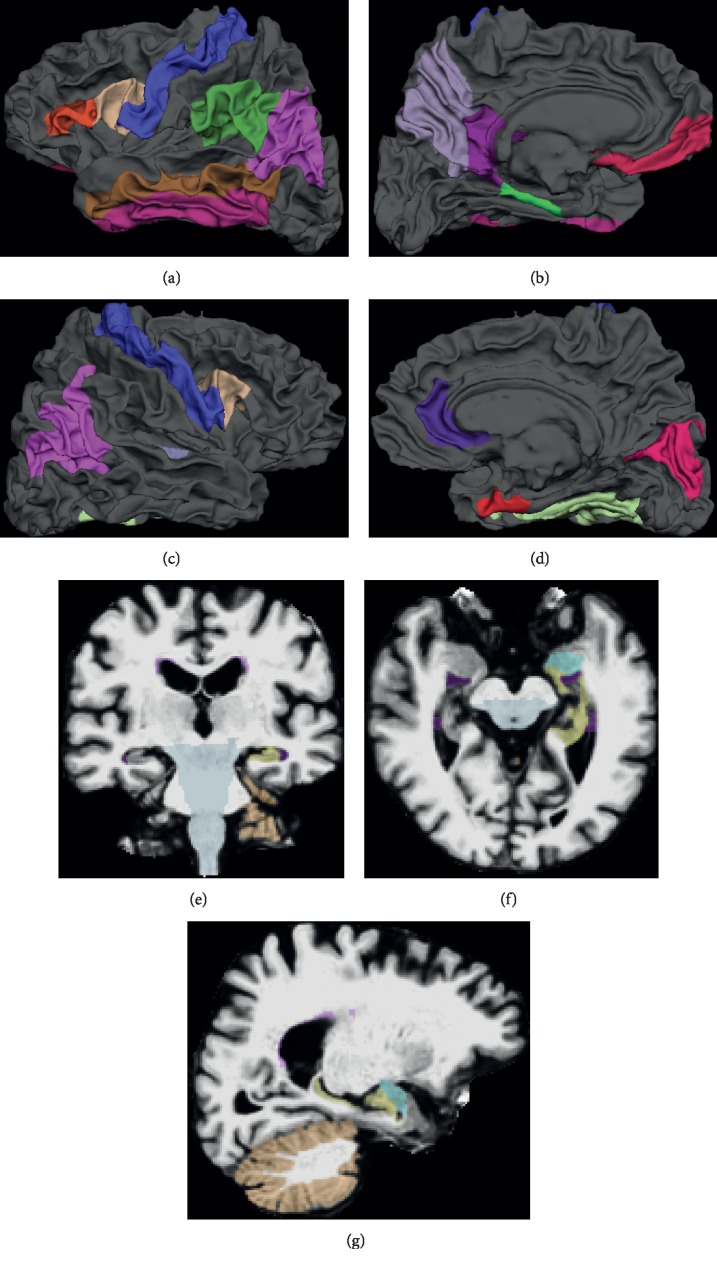
Brain maps of the top 30 ROIs selected by GFL-SGL for MMSE. (a)–(d) are cortical ROIs selected; (e)–(g) are subcortical ROIs selected. (a) Left hemisphere (outside). (b) Left hemisphere (inside). (c) Right hemisphere (outside). (d) Right hemisphere (inside). (e) Coronal view. (f) Horizontal view. (g) Sagittal view.

Moreover, the top 30 selected MRI features and brain regions (ROIs) for ADAS and MMSE are shown in Table 5. We also show the brain maps of the top ROIs in Figures 8 and 9, including cortical ROIs and subcortical ROIs. Note that the top features and ROIs are obtained by calculating the overall weights for the six time points. From the top 30 features, we can examine the group sparsity of GFL-SGL model at the ROI level. It can be seen clearly that many top features come from the same ROI due to the consideration of group property in features, such as L.Hippocampus, L.MidTemporal, L.InfLatVent, and R.Entorhinal.

Some important brain regions are also selected by our GFL-SGL, such as middle temporal [20,40–42], hippocampus [42], entorhinal [20], inferior lateral ventricle [35, 43], and parahipp [44], which are highly relevant to the cognitive impairment. These results are consistent with the established understanding of the pathological pathway of AD. These recognized brain regions have been figured out in the recent literature besides having been presented as have a high correlation with the medical functions. For instance, the hippocampus is situated in the temporal lobe of the brain that plays the part of the memory as well as spatial navigation. The entorhinal cortex refers to the first region of the brain being impacted; also, it is termed as the most severely impaired cortex in Alzheimer's disease [45]. Together with that, there are some of the recent findings stressing the significance of parahippocampal atrophy as a preliminary biomarker of AD, owing to the fact parahippocampal volume makes better discrimination in comparison with the hippocampal volume between the cases of healthy aging, MCI, and mild AD, being specific, in the preliminary stage of the disease [44]. In addition to that, the findings also reveal the fact that the changing thickness of the inferior parietal lobule takes place early while progressing from normal to MCI, together with being associated with the neuropsychological efficiency [46].

### 4.4. Fusion of Multimodality

Clinical and research studies commonly demonstrate that complementary brain images can be more accurate and rigorous for assessment of the disease status and cognitive function. The previous experiments are conduced on the MRI, which measures the structure of the cerebrum and has turned out to be an efficient tool for detecting the structural changes caused by AD or MCI. Fluorodeoxyglucose PET (FDG-PET), a technique for measuring glucose metabolism, can determine the likelihood of deterioration of mental status. Each neuroimaging modality could offer valuable information, and biomarkers from different modalities could offer complementary information for different aspects of a given disease process [4, 14, 47–49].

Since the multimodality data of ADNI-1 are missing seriously, the samples from ADNI-2 are used instead. The PET imaging data are from the ADNI database processed by the UC Berkeley team, who use a native-space MRI scan for each subject that is segmented and parcellated with Freesurfer to generate a summary cortical and subcortical ROI and coregister each florbetapir scan to the corresponding MRI and calculate the mean florbetapir uptake within the cortical and reference regions. The procedure of image processing is described in http://adni.loni.usc.edu/updated-florbetapir-av-45-pet-analysis-results/. The amount of the patients with MRI at M48 is small (29 subjects), and there are no data with PET at M6; 4 time points' data were used. Furthermore, there is no score measure for FLU.ANIM and lack of samples for FLU.VEG and TRAILS, so we use ADAS, MMSE, and RAVLT for a total of 6 scores in this experiment. We followed the same experimental procedure as described in Section 4.1, which yields a total of *n* = 897 subjects for baseline, and for the M12, M24, M36 time points, the sample size is 671, 470, and 62, respectively.

To estimate the effect of combining multimodality data with our GFL-SGL method and to provide a more comprehensive comparison of our group guided method and the method without group structure, we further perform some experiments, which are (1) employing only MRI modality, (2) employing only PET modality, (3) combining two modalities: MRI and PET (MP), and (4) combining four modalities: MRI, PET, CSF, and demographic information including age, gender, years of education, and ApoE genotyping (MPCD). Note that, for the CSF modality, the original three measures (i.e., Aß_42_, *t*-tau, and *p*-tau) are directly used as features without any feature selection step. We compare the performance of TGL, cFSGL, and GFL-SGL on the fusing multimodalities for predicting the disease progression measured by the clinical scores (ADAS-Cog, MMSE, and RAVLT). For TGL and cFSGL, the features from multimodalities are concatenated into long vector features, while for our GFL-SGL, the features from same modality are considered as a group.

The prediction performance results are shown in Table 6. It is clear that the methods with multimodality outperform the methods using one single modality of data. This validates our assumption that the complementary information among different modalities is helpful for cognitive function prediction. Especially, when two modalities (MRI and PET) are used, the performance is improved significantly compared to using the unimodal (MRI or PET) information. Moreover when four modalities (MRI, PET, CSF, and demographic information) are used, the performance is further improved. Regardless of two or four modalities, the proposed multitask learning GFL-SGL achieves better performance than TGL and cFSGL. This justifies the motivation of learning multiple tasks simultaneously with considering the group of variables regardless of the ROI structure or the modality structure.

## 5. Conclusion

In this paper, we investigated the progression of longitudinal Alzheimer's disease (AD) by means of multiple cognitive scores and multimodality data. We proposed a multitask learning formulation with group guided regularization that can exploit the correlation of different time points and the importance of ROIs or multiple modalities for predicting the cognitive scores. Alternating direction method of multipliers (ADMM) method is presented to efficiently tackle the associated optimization problem. Experiments and comparisons of this model, with the baseline and temporal smoothness methods, illustrate that GFL-SGL offers consistently better performance than other algorithms on both MRI features and multimodality data.

In the current work, group guided information is only considered for each cognitive score separately with multiple tasks corresponding to the same cognitive score across multiple time points. And the group guided information used in this work is predefined; there is no ability to automatically learn the feature groups. Since the cognitive scores are used in different ways to measure the same underlying medical condition and the features have different structures, we expect that a more general group guided framework that learns group information automatically will be considered for all cognitive scores across all time points simultaneously. While the current study illustrates the power of our proposed method, we expect to perform more general experiments to validate the effectiveness in our future work. All of the regions processed by UCSF are used in this work. We will consider the medical background and screen these features. In order to compare the significant performance of the methods more effectively, we will randomly split the subjects into train and test. This will be repeated many times to obtain enough scores for statistical analysis.

## Figures and Tables

**Algorithm 1:**
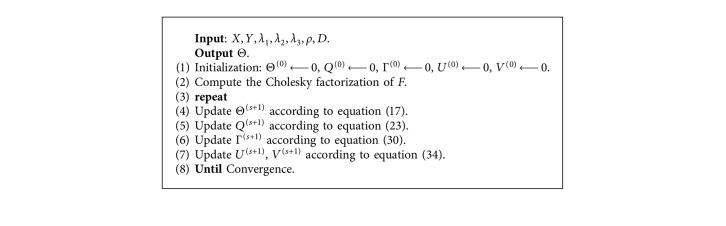
ADMM optimization of GFL-SGL.

**Table 1:** Cortical features from the following 71 (=35 × 2+1) cortical regions generated by FreeSurfer.

ID	ROI name	Laterality	Type
1	Banks superior temporal sulcus	L, R	CV, SA, TA, TS
2	Caudal anterior cingulate cortex	L, R	CV, SA, TA, TS
3	Caudal middle frontal gyrus	L, R	CV, SA, TA, TS
4	Cuneus cortex	L, R	CV, SA, TA, TS
5	Entorhinal cortex	L, R	CV, SA, TA, TS
6	Frontal pole	L, R	CV, SA, TA, TS
7	Fusiform gyrus	L, R	CV, SA, TA, TS
8	Inferior parietal cortex	L, R	CV, SA, TA, TS
9	Inferior temporal gyrus	L, R	CV, SA, TA, TS
10	Insula	L, R	CV, SA, TA, TS
11	Isthmus cingulate	L, R	CV, SA, TA, TS
12	Lateral occipital cortex	L, R	CV, SA, TA, TS
13	Lateral orbital frontal cortex	L, R	CV, SA, TA, TS
14	Lingual gyrus	L, R	CV, SA, TA, TS
15	Medial orbital frontal cortex	L, R	CV, SA, TA, TS
16	Middle temporal gyrus	L, R	CV, SA, TA, TS
17	Paracentral lobule	L, R	CV, SA, TA, TS
18	Parahippocampal gyrus	L, R	CV, SA, TA, TS
19	Pars opercularis	L, R	CV, SA, TA, TS
20	Pars orbitalis	L, R	CV, SA, TA, TS
21	Pars triangularis	L, R	CV, SA, TA, TS
22	Pericalcarine cortex	L, R	CV, SA, TA, TS
23	Postcentral gyrus	L, R	CV, SA, TA, TS
24	Posterior cingulate cortex	L, R	CV, SA, TA, TS
25	Precentral gyrus	L, R	CV, SA, TA, TS
26	Precuneus cortex	L, R	CV, SA, TA, TS
27	Rostral anterior cingulate cortex	L, R	CV, SA, TA, TS
28	Rostral middle frontal gyrus	L, R	CV, SA, TA, TS
29	Superior frontal gyrus	L, R	CV, SA, TA, TS
30	Superior parietal cortex	L, R	CV, SA, TA, TS
31	Superior temporal gyrus	L, R	CV, SA, TA, TS
32	Supramarginal gyrus	L, R	CV, SA, TA, TS
33	Temporal pole	L, R	CV, SA, TA, TS
34	Transverse temporal cortex	L, R	CV, SA, TA, TS
35	Hemisphere	L, R	SA
36	Total intracranial volume	Bilateral	CV

275 (=34 × 2 × 4+1 × 2 × 1+1) cortical features calculated were analyzed in this study. Laterality indicates different feature types calculated for L (left hemisphere), R (right hemisphere), or Bilateral (whole hemisphere).

**Table 2:** Subcortical features from the following 44 (=16 × 2+12) subcortical regions generated by FreeSurfer.

Number	ROI	Laterality	Type
1	Accumbens area	L, R	SV
2	Amygdala	L, R	SV
3	Caudate	L, R	SV
4	Cerebellum cortex	L, R	SV
5	Cerebellum white matter	L, R	SV
6	Cerebral cortex	L, R	SV
7	Cerebral white matter	L, R	SV
8	Choroid plexus	L, R	SV
9	Hippocampus	L, R	SV
10	Inferior lateral ventricle	L, R	SV
11	Lateral ventricle	L, R	SV
12	Pallidum	L, R	SV
13	Putamen	L, R	SV
14	Thalamus	L, R	SV
15	Ventricle diencephalon	L, R	SV
16	Vessel	L, R	SV
17	Brainstem	Bilateral	SV
18	Corpus callosum anterior	Bilateral	SV
19	Corpus callosum central	Bilateral	SV
20	Corpus callosum middle anterior	Bilateral	SV
21	Corpus callosum middle posterior	Bilateral	SV
22	Corpus callosum posterior	Bilateral	SV
23	Cerebrospinal fluid	Bilateral	SV
24	Fourth ventricle	Bilateral	SV
25	Nonwhite matter hypointensities	Bilateral	SV
26	Optic chiasm	Bilateral	SV
27	Third ventricle	Bilateral	SV
28	White matter hypointensities	Bilateral	SV

44 subcortical features calculated were analyzed in this study. Laterality indicates different feature types calculated for L (left hemisphere), R (right hemisphere), or Bilateral (whole hemisphere).

**Table 3:** Description of the cognitive scores considered in the experiments.

Score name		Description
ADAS		Alzheimer's disease assessment scale
MMSE		Mini-mental state exam
RAVLT	TOTAL	Total score of the first 5 learning trials
	TOT6	Trial 6 total number of words recalled
	T30	30 minute delay total number of words recalled
	RECOG	30 minute delay recognition
FLU	ANIM	Animal total score
	VEG	Vegetable total score
TRAILS	A	Trail making test A score
	B	Trail making test B score

**Table 4:** Prediction performance results of ten cognitive scores of six time points based on MRI features.

	Ridge	Lasso	TGL	cFSGL	GFL-SGL
*Score: ADAS*					
nMSE	10.122 ± 1.4156^*∗*^	6.7689 ± 0.7698^*∗*^	6.2740 ± 0.7861	6.3092 ± 0.6991^*∗*^	**6.1389** **±** **0.6951**
wR	0.5638 ± 0.0509^*∗*^	0.6237 ± 0.0541^*∗*^	0.6628 ± 0.0561	0.6560 ± 0.0486^*∗*^	**0.6658** **±** **0.0469**
BL rMSE	7.6553 ± 0.5576	6.8217 ± 0.4238	6.7275 ± 0.4298	6.7151 ± 0.4275	**6.6479** **±** **0.5045**
M6 rMSE	9.1778 ± 1.2467	7.9602 ± 0.8484	7.7637 ± 0.9345	**7.6846** **±** **0.9493**	7.6994 **±** 0.9357
M12 rMSE	9.7212 ± 1.0986	8.7050 ± 0.8651	**8.3822** **±** **0.9401**	8.4646 ± 1.0594	8.4076 ± 1.0478
M24 rMSE	11.676 ± 1.6463	10.191 ± 1.2914	9.6773 ± 1.6308	9.7859 ± 1.6170	**9.4808** **±** **1.6224**
M36 rMSE	12.772 ± 2.4262	9.4852 ± 1.3806	8.9110 ± 1.3356	8.9313 ± 1.3762	**8.7939** **±** **1.2987**
M48 rMSE	20.433 ± 2.6163	9.0161 ± 2.3381	8.2041 ± 1.1869	8.6279 ± 2.0852	**8.0947** **±** **1.6669**

*Score: MMSE*					
nMSE	10.447 ± 1.4590^*∗*^	2.5284 ± 0.2230^*∗*^	2.4911 ± 0.1411^*∗*^	2.5048 ± 0.1772^*∗*^	**2.3975** **±** **0.2140**
wR	0.4188 ± 0.0530^*∗*^	0.5720 ± 0.0498^*∗*^	0.5898 ± 0.0431^*∗*^	0.5878 ± 0.0449^*∗*^	**0.5975** **±** **0.0425**
BL rMSE	2.6943 ± 0.1767	2.2001 ± 0.1349	2.2204 ± 0.1367	2.1729 ± 0.1505	**2.1478** **±** **0.1159**
M6 rMSE	3.5136 ± 0.3413	2.8571 ± 0.2697	2.8260 ± 0.2875	2.8069 ± 0.2882	**2.7682** **±** **0.2470**
M12 rMSE	3.9044 ± 0.2313	3.2128 ± 0.3301	3.1438 ± 0.3328	3.1558 ± 0.3650	**3.1375** **±** **0.3660**
M24 rMSE	5.0192 ± 0.6956	3.8663 ± 0.6975	**3.8171** **±** **0.7064**	3.8316 ± 0.6355	3.8371 ± 0.7620
M36 rMSE	5.7022 ± 0.5505	3.2518 ± 0.8592	3.2732 ± 0.8106	3.4828 ± 0.6365	**3.1914** **±** **0.8230**
M48 rMSE	29.958 ± 0.7233	4.0539 ± 0.7097	4.0077 ± 0.8089	3.8018 ± 0.9474	**3.5517** **±** **0.6933**

*Score: RAVLT.TOTAL*					
nMSE	17.139 ± 1.2384^*∗*^	9.7932 ± 0.9119^*∗*^	9.1381 ± 0.8168^*∗*^	8.9621 ± 0.9867	**8.7825** **±** **0.9241**
wR	0.4059 ± 0.0510^*∗*^	0.4989 ± 0.0587^*∗*^	0.5390 ± 0.0603	0.5498 ± 0.0533	**0.5512** **±** **0.0558**
BL rMSE	11.404 ± 0.7043	9.8789 ± 0.9286	9.6628 ± 0.9091	9.6980 ± 0.7418	**9.5445** **±** **0.6940**
M6 rMSE	11.828 ± 1.1623	10.210 ± 1.2512	9.9696 ± 1.1915	10.079 ± 1.1682	**9.8337** **±** **1.2773**
M12 rMSE	13.027 ± 0.9974	11.457 ± 0.9096	10.945 ± 1.1063	10.865 ± 1.3290	**10.788** **±** **1.2737**
M24 rMSE	14.647 ± 1.4006	12.330 ± 1.4231	11.997 ± 1.5765	11.756 ± 1.6851	**11.740** **±** **1.5374**
M36 rMSE	15.899 ± 2.2567	11.512 ± 1.5268	10.640 ± 1.2792	10.331 ± 1.5089	**10.306** **±** **1.5535**
M48 rMSE	41.462 ± 3.4404	12.728 ± 1.5048	13.105 ± 2.8874	11.333 ± 2.0937	**11.803** **±** **2.5033**

*Score: RAVLT.TOT6*					
nMSE	3.9829 ± 0.4397^*∗*^	2.9663 ± 0.1909^*∗*^	2.8853 ± 0.2057^*∗*^	2.8546 ± 0.1867	**2.8198** **±** **0.1772**
wR	0.4528 ± 0.0703^*∗*^	0.5213 ± 0.0803^*∗*^	0.5412 ± 0.0682^*∗*^	0.5458 ± 0.0687^*∗*^	**0.5541** **±** **0.0730**
BL rMSE	3.6885 ± 0.3741	3.2944 ± 0.2617	3.2949 ± 0.2611	3.2756 ± 0.2885	**3.2540** **±** **0.2390**
M6 rMSE	3.4704 ± 0.3949	3.1592 ± 0.3443	3.1628 ± 0.2939	3.1386 ± 0.3116	**3.1270** **±** **0.2841**
M12 rMSE	3.8384 ± 0.2676	3.4284 ± 0.2262	3.4271 ± 0.2632	3.4094 ± 0.2808	**3.3763** **±** **0.2575**
M24 rMSE	4.0656 ± 0.3758	3.6252 ± 0.3469	3.5826 ± 0.3581	3.5894 ± 0.3360	**3.5592** **±** **0.3293**
M36 rMSE	4.3074 ± 0.7174	3.5169 ± 0.3667	3.3890 ± 0.3799	3.3799 ± 0.3926	**3.3557** **±** **0.3799**
M48 rMSE	7.4599 ± 1.0656	4.5834 ± 0.6969	3.7902 ± 0.7846	**3.7275** **±** **0.7056**	3.7694 ± 0.7746

*Score: RAVLT.T30*					
nMSE	3.9392 ± 0.3946^*∗*^	3.0595 ± 0.2012^*∗*^	2.9876 ± 0.1950	2.9706 ± 0.2044	**2.9358** **±** **0.1919**
wR	0.4580 ± 0.0609^*∗*^	0.5255 ± 0.0730^*∗*^	0.5384 ± 0.0679	0.5422 ± 0.0647	**0.5474** **±** **0.0646**
BL rMSE	3.7877 ± 0.3069	3.4076 ± 0.2595	3.4176 ± 0.2485	3.4034 ± 0.2806	**3.3806** **±** **0.2491**
M6 rMSE	3.4750 ± 0.3531	3.1839 ± 0.3380	3.2095 ± 0.2593	3.1991 ± 0.2871	**3.1496** **±** **0.3013**
M12 rMSE	3.9611 ± 0.4480	3.6673 ± 0.3242	3.6343 ± 0.3799	3.6173 ± 0.3800	**3.5943** **±** **0.3790**
M24 rMSE	4.2027 ± 0.5011	3.8070 ± 0.4648	3.7570 ± 0.4051	3.7562 ± 0.4151	**3.7389** **±** **0.4429**
M36 rMSE	4.2142 ± 0.5102	3.5049 ± 0.4595	3.3604 ± 0.4241	**3.3473** **±** **0.4327**	3.3852 ± 0.4545
M48 rMSE	7.1834 ± 0.8145	4.5537 ± 0.6315	**4.0102** **±** **0.4413**	4.0900 ± 0.5064	4.0727 ± 0.5386

*Score: RAVLT.RECOG*					
nMSE	6.2754 ± 1.2306^*∗*^	3.4921 ± 0.3325^*∗*^	**3.2186** **±** **0.2953**	3.2282 ± 0.2992	3.2314 ± 0.2654
wR	0.3496 ± 0.0851^*∗*^	0.4583 ± 0.0793^*∗*^	0.4993 ± 0.0779	**0.5075** **±** **0.0738**	0.5058 ± 0.0799
BL rMSE	4.3887 ± 0.4210	3.6494 ± 0.2993	3.5990 ± 0.3647	3.5721 ± 0.3709	**3.5653** **±** **0.3386**
M6 rMSE	4.4959 ± 0.3686	3.7470 ± 0.2412	3.6722 ± 0.2928	**3.6616** **±** **0.2995**	3.6627 ± 0.2815
M12 rMSE	4.6874 ± 0.3574	3.7850 ± 0.2889	3.7034 ± 0.3521	3.7178 ± 0.2935	**3.6942** **±** **0.3141**
M24 rMSE	4.8253 ± 0.4029	3.9168 ± 0.2251	**3.7518** **±** **0.2771**	3.8103 ± 0.2391	3.8058 ± 0.2488
M36 rMSE	5.4178 ± 0.6548	3.8073 ± 0.2366	**3.6448** **±** **0.2966**	3.6962 ± 0.1616	3.7372 ± 0.1837
M48 rMSE	12.411 ± 0.9035	5.1582 ± 1.0963	3.9023 ± 0.8880	**3.7995** **±** **0.8998**	3.9423 ± 0.7025

*Score: FLU.ANIM*					
nMSE	9.6435 ± 1.1387^*∗*^	5.2513 ± 0.7213^*∗*^	5.1293 ± 0.6597^*∗*^	4.9992 ± 0.6243	**4.9478** **±** **0.6151**
wR	0.2872 ± 0.0942^*∗*^	0.3858 ± 0.0834^*∗*^	0.4212 ± 0.0895^*∗*^	**0.4433** **±** **0.0839**	0.4406 ± 0.0840
BL rMSE	6.3878 ± 0.6423	5.2970 ± 0.5354	5.3535 ± 0.4841	**5.1972** **±** **0.5149**	5.2026 ± 0.4857
M6 rMSE	6.1380 ± 0.5975	5.3040 ± 0.4995	5.3207 ± 0.4732	5.2175 ± 0.4797	**5.1951** **±** **0.4563**
M12 rMSE	6.6219 ± 0.7800	5.7413 ± 0.8672	5.6134 ± 0.7977	5.5704 ± 0.8052	**5.5303** **±** **0.7929**
M24 rMSE	7.2828 ± 0.9366	5.8387 ± 0.7492	5.7844 ± 0.6280	5.7839 ± 0.7570	**5.6815** **±** **0.7035**
M36 rMSE	7.8427 ± 1.4361	5.6450 ± 0.6733	**5.3599** **±** **0.7423**	5.3988 ± 0.8188	5.3655 ± 0.6841
M48 rMSE	20.613 ± 1.8524	6.2549 ± 1.6986	**5.7005** **±** **1.3167**	5.7501 ± 1.5019	5.9240 ± 1.4382

*Score: FLU.VEG*					
nMSE	6.6621 ± 0.8499^*∗*^	3.5364 ± 0.3463^*∗*^	3.4061 ± 0.2879	3.3593 ± 0.3146	3.3575 ± 0.2867
wR	0.3726 ± 0.0730^*∗*^	0.4934 ± 0.0830^*∗*^	0.5257 ± 0.0781	**0.5357** **±** **0.0746**	0.5356 ± 0.0777
BL rMSE	4.4121 ± 0.3082	3.7115 ± 0.2221	3.6980 ± 0.2387	3.6464 ± 0.2179	**3.6368** **±** **0.2016**
M6 rMSE	4.7036 ± 0.1969	3.8593 ± 0.2589	3.8617 ± 0.2075	3.8033 ± 0.2318	**3.7892** **±** **0.2294**
M12 rMSE	5.0566 ± 0.4772	3.9568 ± 0.4941	3.9319 ± 0.4757	**3.9226** **±** **0.4542**	3.9267 ± 0.4761
M24 rMSE	5.2146 ± 0.4402	4.2580 ± 0.4104	**4.1408** **±** **0.3444**	4.1677 ± 0.4192	4.1908 ± 0.4275
M36 rMSE	6.4334 ± 0.7933	4.4230 ± 0.3982	4.2656 ± 0.3702	4.2445 ± 0.4263	4.2392 ± 0.3829
M48 rMSE	13.882 ± 1.4535	4.9607 ± 1.4253	**3.9822** **±** **1.3527**	3.9887 ± 1.4023	4.0292 ± 1.4371

*Score: TRAILS.A*					
nMSE	33.513 ± 3.8491^*∗*^	23.711 ± 1.8805	**22.756** **±** **1.5155**	23.151 ± 1.5754	23.349 ± 1.5768
wR	0.3572 ± 0.0769^*∗*^	0.3740 ± 0.0658^*∗*^	**0.4219** **±** **0.0682**	0.4122 ± 0.0688	0.3965 ± 0.0704
BL rMSE	25.942 ± 3.8665	23.421 ± 4.0061	**23.039** **±** **3.6598**	23.258 ± 3.7233	23.443 ± 3.8347
M6 rMSE	28.290 ± 4.4832	25.328 ± 3.6847	**25.021** **±** **3.3715**	25.198 ± 3.5600	25.634 ± 3.3660
M12 rMSE	27.665 ± 3.8961	25.043 ± 3.4997	**24.493** **±** **3.3011**	24.675 ± 3.3022	24.882 ± 3.2310
M24 rMSE	31.805 ± 4.1087	28.384 ± 3.0384	**27.845** **±** **3.2106**	28.073 ± 3.1074	27.855 ± 3.0427
M36 rMSE	33.414 ± 8.1383	24.980 ± 7.0999	**23.996** **±** **5.2222**	24.162 ± 5.9112	24.247 ± 6.0955
M48 rMSE	53.906 ± 14.730	28.256 ± 16.054	**26.493** **±** **12.132**	26.870 ± 11.598	25.241 ± 11.862

*Score: TRAILS.B*					
nMSE	94.882 ± 9.6015^*∗*^	68.077 ± 6.4277^*∗*^	64.789 ± 5.9269	63.707 ± 6.2629	**63.604** **±** **5.5813**
wR	0.3837 ± 0.0509^*∗*^	0.4383 ± 0.0618^*∗*^	0.4845 ± 0.0565	0.4809 ± 0.0669	**0.4858** **±** **0.0595**
BL rMSE	77.907 ± 6.5622	70.051 ± 4.5144	69.947 ± 4.9343	**69.032** **±** **3.8304**	69.154 ± 4.0030
M6 rMSE	83.326 ± 7.1076	74.327 ± 4.2985	72.514 ± 3.4677	**71.401** **±** **4.4814**	71.756 ± 4.9096
M12 rMSE	81.130 ± 8.9465	72.901 ± 6.0166	**70.604** **±** **5.8510**	70.777 ± 6.4053	70.815 ± 5.7209
M24 rMSE	89.969 ± 13.035	77.722 ± 8.9225	**73.456** **±** **9.3979**	73.950 ± 8.5186	73.460 ± 9.2281
M36 rMSE	100.25 ± 21.732	80.934 ± 26.923	78.130 ± 24.536	78.242 ± 27.867	**77.639** **±** **23.797**
M48 rMSE	134.89 ± 29.881	67.923 ± 29.604	68.356 ± 11.968	65.858 ± 24.964	**63.491** **±** **18.188**

Note that the best results are boldfaced. The superscript symbol “^*∗*^” indicates that GFL-SGL significantly outperformed that method on that score. Paired *t*-test at a level of 0.05 was used.

**Table 5:** Top 30 selected MRI features and ROIs by GFL-SGL on the prediction ADAS and MMSE measures.

Num.	ADAS		MMSE	
	Features	Groups	Features	Groups
1	SV of L.HippVol	L.Hippocampus	SV of L.HippVol	L.Hippocampus
2	TA of L.MidTemporal	L.MidTemporal	TA of L.MidTemporal	L.InfLatVent
3	TA of R.Entorhinal	L.InfLatVent	TA of R.Entorhinal	L.MidTemporal
4	CV of R.Entorhinal	R.Entorhinal	CV of R.Entorhinal	R.Entorhinal
5	SV of L.InfLatVent	L.CerebellCtx	SV of L.InfLatVent	R.InfLatVent
6	SV of L.CerebellCtx	L.Thalamus	CV of L.MidTemporal	L.InfParietal
7	TA of L.InfTemporal	L.Pallidum	TA of L.InfParietal	CC_Ant
8	TS of L.Parahipp	CC_Ant	SV of R.InfLatVent	WMHypoInt
9	TA of R.InfParietal	R.InfParietal	TS of L.Parahipp	R.InfParietal
10	CV of L.Precentral	L.Precentral	TS of R.RostAntCing	L.Parahipp
11	TA of L.Precuneus	L.InfTemporal	TA of R.InfParietal	R.RostAntCing
12	SV of L.ThalVol	L.Precuneus	CV of L.InfParietal	Brainstem
13	TS of L.ParsTriang	R.Precentral	SV of CC_Ant	L.Supramarg
14	SV of L.PallVol	L.Parahipp	CV of R.InfParietal	R.Precentral
15	CV of R.Precentral	L.ParsTriang	SV of WMHypoInt	4thVent
16	SA of L.Supramarg	L.Supramarg	TA of R.TransvTemporal	R.TransvTemporal
17	TA of L.Postcentral	L.Postcentral	TS of L.InfParietal	R.BanksSTS
18	CV of R.InfParietal	CSF	SA of L.Supramarg	L.InfTemporal
19	SV of CC_Ant	OpticChiasm	CV of L.Supramarg	L.Precentral
20	SA of L.RostAntCing	L.TemporalPole	SA of R.InfParietal	R.Cuneus
21	TA of R.Precentral	R.TransvTemporal	SA of L.MidTemporal	L.Amygdala
22	CV of L.TemporalPole	L.LatOrbFrontal	TA of L.Parahipp	OpticChiasm
23	CV of L.LatOrbFrontal	L.RostAntCing	TA of R.Precentral	L.MedOrbFrontal
24	CV of R.TransvTemporal	R.CerebWM	TA of L.InfTemporal	L.IsthmCing
25	TS of L.SupFrontal	R.SupParietal	SA of L.Parahipp	L.ParsOper
26	TS of R.Parahipp	L.SupFrontal	CV of R.Precentral	L.CerebellCtx
27	CV of R.SupParietal	R.AccumbensArea	CV of R.TransvTemporal	L.ParsTriang
28	CV of L.MidTemporal	R.Cuneus	TA of R.BanksSTS	R.ParsOper
29	TS of L.Precentral	3rdVent	SA of L.InfParietal	L.Precuneus
30	TS of L.InfTemporal	L.IsthmCing	CV of L.Precentral	R.Fusiform

**Table 6:** Prediction performance results of ten cognitive scores of four time points based on multimodality features.

Method	TGL	cFSGL	GFL-SGL	TGL	cFSGL	GFL-SGL
*Score: ADAS*						
	MRI			PET		
nMSE	4.5264 ± 0.6382^*∗*^	4.4109 ± 0.5918^*∗*^	4.6987 ± 0.7419^*∗*^	4.6438 ± 0.6733^*∗*^	4.4061 ± 0.6413^*∗*^	4.4294 ± 0.6974^*∗*^
wR	0.6806 ± 0.0877^*∗*^	0.6806 ± 0.0853^*∗*^	0.6692 ± 0.0897^*∗*^	0.6792 ± 0.0716^*∗*^	0.6940 ± 0.0755^*∗*^	0.6997 ± 0.0842^*∗*^
BL rMSE	6.3971 ± 1.1270	6.3670 ± 1.0509	6.5227 ± 1.3104	6.5252 ± 1.4146	6.3755 ± 1.3076	6.3614 ± 1.3953
M12 rMSE	5.8845 ± 1.1703	5.8519 ± 1.0680	6.0360 ± 1.1920	6.0747 ± 1.2560	5.8881 ± 1.0507	5.8035 ± 1.0189
M24 rMSE	5.2531 ± 0.9237	5.2829 ± 0.9966	5.4175 ± 0.9846	5.4755 ± 1.0622	5.2970 ± 1.0386	5.2901 ± 0.9836
M36 rMSE	5.6362 ± 1.4445	4.5437 ± 1.5677	5.0457 ± 1.8708	4.4315 ± 1.8231	4.2938 ± 1.5977	5.0727 ± 2.0042

*Score: ADAS*						
	MP			MPCD		
nMSE	4.3771 ± 0.8225^*∗*^	4.0380 ± 0.5282^*∗*^	3.8140 ± 0.7056^*∗*^	4.1169 ± 0.5791^*∗*^	3.9251 ± 0.4846^*∗*^	**3.7255** **±** **0.6441**
wR	0.7140 ± 0.0756^*∗*^	0.7178 ± 0.0717^*∗*^	0.7400 ± 0.0942	0.7222 ± 0.0633^*∗*^	0.7267 ± 0.0633^*∗*^	**0.7477** **±** **0.0842**
BL rMSE	6.1640 ± 1.0841	6.1365 ± 1.0937	5.9200 ± 1.0238	6.0910 ± 1.1327	6.0447 ± 1.1147	**5.8632** **±** **1.0630**
M12 rMSE	5.6180 ± 0.9929	5.5713 ± 0.9659	5.2731 ± 0.7940	5.5036 ± 1.0018	5.5110 ± 0.9877	**5.2172** **±** **0.7874**
M24 rMSE	5.3149 ± 0.9609	5.0187 ± 0.9873	4.7865 ± 0.7474	5.1299 ± 1.0265	4.9841 ± 1.0004	**4.7442** **±** **0.7253**
M36 rMSE	6.1291 ± 1.7931	4.3765 ± 1.5989	5.1638 ± 1.6369	5.4648 ± 1.8165	**4.2363** **±** **1.2992**	5.0341 ± 1.5739

*Score: MMSE*						
	MRI			PET		
nMSE	1.9059 ± 0.3673^*∗*^	1.5544 ± 0.1589^*∗*^	1.5446 ± 0.1709^*∗*^	2.0863 ± 0.9497^*∗*^	1.8916 ± 0.4145^*∗*^	1.5699 ± 0.1326^*∗*^
wR	0.4737 ± 0.1132^*∗*^	0.5449 ± 0.1009^*∗*^	0.5383 ± 0.1014^*∗*^	0.4988 ± 0.0906^*∗*^	0.5233 ± 0.0813^*∗*^	0.5270 ± 0.0843^*∗*^
BL rMSE	1.9866 ± 0.2782	1.8715 ± 0.3253	1.9085 ± 0.3409	2.0294 ± 0.3362	1.9772 ± 0.3649	1.8974 ± 0.3600
M12 rMSE	1.9969 ± 0.4054	1.7781 ± 0.2863	1.7843 ± 0.2764	1.9950 ± 0.4867	1.9040 ± 0.2725	1.8229 ± 0.2912
M24 rMSE	1.8220 ± 0.4220	1.6044 ± 0.2843	1.5656 ± 0.3140	1.9738 ± 0.7546	1.7230 ± 0.2846	1.5950 ± 0.2976
M36 rMSE	1.9900 ± 1.0439	1.6339 ± 0.5279	1.4005 ± 0.4729	1.9142 ± 0.8379	2.3950 ± 2.0477	1.3472 ± 0.4827

*Score: MMSE*						
	MP			MPCD		
nMSE	1.7323 ± 0.3153^*∗*^	1.5056 ± 0.1055^*∗*^	1.4386 ± 0.1310^*∗*^	1.7428 ± 0.4059^*∗*^	1.5697 ± 0.2846^*∗*^	**1.3881** **±** **0.1132**
wR	0.5128 ± 0.0950^*∗*^	0.5763 ± 0.0969^*∗*^	0.5743 ± 0.0996^*∗*^	0.5352 ± 0.0931^*∗*^	**0.5961** **±** **0.1005**	0.5899 ± 0.0882
BL rMSE	1.9714 ± 0.3393	1.8456 ± 0.3451	1.8486 ± 0.3119	1.9487 ± 0.3099	1.8780 ± 0.3308	**1.8185** **±** **0.3082**
M12 rMSE	1.8040 ± 0.2961	1.7277 ± 0.2223	1.7144 ± 0.2366	1.8753 ± 0.4289	1.7615 ± 0.2122	**1.6804** **±** **0.2269**
M24 rMSE	1.7497 ± 0.4408	1.5849 ± 0.2728	1.4954 ± 0.2847	1.7516 ± 0.4781	1.5927 ± 0.2903	**1.4702** **±** **0.2631**
M36 rMSE	1.8481 ± 0.8608	1.5549 ± 0.5338	1.3110 ± 0.3568	1.6768 ± 0.8175	1.5542 ± 0.5532	**1.2835** **±** **0.4067**

*Score: RAVLT.TOTAL*						
	MRI			PET		
nMSE	8.0525 ± 0.8185^*∗*^	7.7486 ± 0.8179^*∗*^	7.6082 ± 0.6860^*∗*^	7.9924 ± 0.5839^*∗*^	7.8193 ± 0.8231^*∗*^	7.6544 ± 0.7192^*∗*^
wR	0.5989 ± 0.0863^*∗*^	0.6094 ± 0.0843^*∗*^	0.6091 ± 0.0790^*∗*^	0.6060 ± 0.0847^*∗*^	0.6003 ± 0.0879^*∗*^	0.6114 ± 0.0853^*∗*^
BL rMSE	9.9809 ± 0.4439	9.8006 ± 0.4509	9.7251 ± 0.4688	9.8305 ± 0.5759	9.7743 ± 0.5324	9.6843 ± 0.5663
M12 rMSE	9.8284 ± 0.6685	9.6394 ± 0.7234	9.5819 ± 0.7833	9.8347 ± 0.7366	9.7590 ± 0.8969	9.6180 ± 0.8630
M24 rMSE	9.4549 ± 0.7243	9.2849 ± 0.7749	9.2384 ± 0.6425	9.8301 ± 0.9371	9.4391 ± 0.9715	9.4183 ± 0.9896
M36 rMSE	9.3364 ± 2.2043	8.9823 ± 1.7996	8.5631 ± 1.9444	7.8316 ± 3.0020	8.6391 ± 2.9697	8.3481 ± 2.5665

*Score: RAVLT.TOTAL*						
	MP			MPCD		
nMSE	7.4966 ± 0.9717^*∗*^	7.2046 ± 0.8326^*∗*^	7.0655 ± 1.1493	7.1461 ± 0.8736^*∗*^	6.7422 ± 1.0602	**6.6873** **±** **0.6733**
wR	0.6350 ± 0.0878^*∗*^	0.6474 ± 0.0888^*∗*^	0.6484 ± 0.0880^*∗*^	0.6617 ± 0.0839	**0.6785** **±** **0.0777**	0.6749 ± 0.0631
BL rMSE	9.6097 ± 0.5299	9.4845 ± 0.4346	9.4516 ± 0.6709	9.4001 ± 0.4595	**9.1793** **±** **0.4505**	9.2208 ± 0.5886
M12 rMSE	9.6195 ± 0.8968	9.2463 ± 0.8042	9.1269 ± 0.8163	9.3194 ± 0.8020	8.8950 ± 0.8305	**8.8526** **±** **0.6871**
M24 rMSE	9.1631 ± 1.0479	8.9473 ± 0.9751	8.7158 ± 0.9721	9.0133 ± 1.1389	8.7459 ± 1.2098	**8.5530** **±** **0.8763**
M36 rMSE	7.7625 ± 2.4338	8.2418 ± 2.4518	7.9290 ± 2.6269	**7.5097** **±** **2.0572**	7.5204 ± 1.9360	7.8103 ± 1.9563

*Score: RAVLT.TOT6*						
	MRI			PET		
nMSE	2.8868 ± 0.2822^*∗*^	2.6401 ± 0.3055^*∗*^	2.6064 ± 0.2727^*∗*^	2.8255 ± 0.2411^*∗*^	2.7979 ± 0.3045^*∗*^	2.6485 ± 0.2487^*∗*^
wR	0.5500 ± 0.0947^*∗*^	0.5910 ± 0.0924^*∗*^	0.5944 ± 0.0903^*∗*^	0.5577 ± 0.0894^*∗*^	0.5599 ± 0.0931^*∗*^	0.5890 ± 0.0806^*∗*^
BL rMSE	3.4094 ± 0.2020	3.2999 ± 0.1986	3.2783 ± 0.1890	3.3321 ± 0.1612	3.3348 ± 0.2227	3.2591 ± 0.2057
M12 rMSE	3.3799 ± 0.2517	3.2278 ± 0.2236	3.2090 ± 0.2246	3.3612 ± 0.1926	3.3499 ± 0.2158	3.2439 ± 0.2019
M24 rMSE	3.3710 ± 0.2493	3.1341 ± 0.3127	3.1229 ± 0.2896	3.3609 ± 0.3845	3.2712 ± 0.3592	3.2008 ± 0.3248
M36 rMSE	3.1387 ± 0.9838	2.9726 ± 0.8134	2.9418 ± 0.8377	3.1407 ± 0.5365	3.2738 ± 0.5865	3.1430 ± 0.6435

*Score: RAVLT.TOT6*						
	MP			MPCD		
nMSE	2.7875 ± 0.3857^*∗*^	2.4537 ± 0.3621	2.4498 ± 0.3377	2.6628 ± 0.3559^*∗*^	2.4224 ± 0.3332^*∗*^	**2.3788** **±** **0.3278**
wR	0.5778 ± 0.1049^*∗*^	0.6233 ± 0.0978	0.6234 ± 0.0970^*∗*^	0.5975 ± 0.0924^*∗*^	0.6322 ± 0.0941^*∗*^	**0.6385** **±** **0.0945**
BL rMSE	3.3512 ± 0.1991	3.1895 ± 0.2325	3.1879 ± 0.2241	3.2930 ± 0.1280	3.1716 ± 0.1708	**3.1401** **±** **0.1625**
M12 rMSE	3.3006 ± 0.2932	3.1072 ± 0.2349	3.0973 ± 0.2367	3.2311 ± 0.3066	3.0928 ± 0.2264	**3.0549** **±** **0.2382**
M24 rMSE	3.2935 ± 0.3180	2.9931 ± 0.3542	2.9989 ± 0.3644	3.2161 ± 0.3336	2.9747 ± 0.3850	**2.9621** **±** **0.3640**
M36 rMSE	3.1544 ± 0.9584	2.8170 ± 0.6229	2.8485 ± 0.6632	2.8216 ± 0.9254	**2.7400** **±** **0.5987**	2.7649 ± 0.6387

*Score: RAVLT.T30*						
	MRI			PET		
nMSE	3.0202 ± 0.2655^*∗*^	2.8297 ± 0.3403^*∗*^	2.7928 ± 0.3498^*∗*^	2.9929 ± 0.4670^*∗*^	2.9441 ± 0.4039^*∗*^	2.9405 ± 0.4265^*∗*^
wR	0.5590 ± 0.0615^*∗*^	0.5861 ± 0.0742^*∗*^	0.5930 ± 0.0721^*∗*^	0.5551 ± 0.0787^*∗*^	0.5692 ± 0.0808^*∗*^	0.5663 ± 0.0780^*∗*^
BL rMSE	3.5917 ± 0.2374	3.5116 ± 0.2514	3.4962 ± 0.2530	3.5711 ± 0.2889	3.5457 ± 0.3032	3.5681 ± 0.3175
M12 rMSE	3.5622 ± 0.2249	3.4155 ± 0.2275	3.3880 ± 0.2550	3.4596 ± 0.3294	3.4806 ± 0.2565	3.4492 ± 0.2890
M24 rMSE	3.5364 ± 0.2177	3.3545 ± 0.2571	3.3244 ± 0.2403	3.5668 ± 0.3435	3.4654 ± 0.3094	3.4536 ± 0.3149
M36 rMSE	2.8940 ± 1.0197	2.8383 ± 1.1163	2.8168 ± 1.1970	3.2296 ± 1.0962	3.2096 ± 1.0170	3.2185 ± 1.1173

*Score: RAVLT.T30*						
	MP			MPCD		
nMSE	2.8661 ± 0.4574^*∗*^	2.6653 ± 0.4271	2.6191 ± 0.4000	2.8335 ± 0.4235^*∗*^	2.6732 ± 0.5128	**2.5605** **±** **0.3929**
wR	0.5811 ± 0.0777^*∗*^	0.6213 ± 0.0828	0.6241 ± 0.0806	0.5955 ± 0.0752^*∗*^	0.6229 ± 0.0907	**0.6369** **±** **0.0820**
BL rMSE	3.5165 ± 0.2941	3.4327 ± 0.2917	3.4035 ± 0.2899	3.4891 ± 0.2710	3.4373 ± 0.2959	**3.3650** **±** **0.2547**
M12 rMSE	3.4078 ± 0.3479	3.3028 ± 0.2590	3.2698 ± 0.2802	3.4073 ± 0.3256	3.3030 ± 0.3404	**3.2280** **±** **0.2819**
M24 rMSE	3.4425 ± 0.2763	3.1974 ± 0.3307	3.1882 ± 0.2826	3.4122 ± 0.2822	3.1894 ± 0.4132	**3.1638** **±** **0.3322**
M36 rMSE	2.9862 ± 1.1763	2.7979 ± 1.0262	2.7246 ± 1.0379	2.9526 ± 1.1659	2.7488 ± 1.0675	**2.6291** **±** **0.9136**

*Score: RAVLT.RECOG*						
	MRI			PET		
nMSE	2.7632 ± 0.2634^*∗*^	2.6598 ± 0.2204^*∗*^	2.6003 ± 0.3379^*∗*^	2.8033 ± 0.5374^*∗*^	2.6547 ± 0.4051^*∗*^	2.6324 ± 0.4138^*∗*^
wR	0.4662 ± 0.1014^*∗*^	0.4728 ± 0.1037^*∗*^	0.5035 ± 0.1158^*∗*^	0.4830 ± 0.1530^*∗*^	0.4955 ± 0.1426^*∗*^	0.5053 ± 0.1311^*∗*^
BL rMSE	3.1427 ± 0.2787	3.1581 ± 0.3301	3.0909 ± 0.3185	3.1383 ± 0.3398	3.0983 ± 0.3626	3.0782 ± 0.3718
M12 rMSE	3.0770 ± 0.4168	3.0286 ± 0.3795	2.9733 ± 0.3981	3.0581 ± 0.4946	2.9974 ± 0.3816	2.9664 ± 0.3816
M24 rMSE	2.9060 ± 0.3237	2.7350 ± 0.2852	2.7274 ± 0.2901	2.9088 ± 0.2768	2.7838 ± 0.3548	2.7737 ± 0.3809
M36 rMSE	2.5846 ± 0.7415	2.2824 ± 0.5062	2.4006 ± 0.5876	2.7997 ± 0.7357	2.5641 ± 0.3984	2.5969 ± 0.8513

*Score: RAVLT.RECOG*						
	MP			MPCD		
nMSE	2.7116 ± 0.3045^*∗*^	2.5529 ± 0.4180	2.4729 ± 0.3549	2.7455 ± 0.2660^*∗*^	2.5057 ± 0.3914	**2.4583** **±** **0.3645**
wR	0.4849 ± 0.1114^*∗*^	0.5279 ± 0.1193	0.5376 ± 0.1176	0.4926 ± 0.1136^*∗*^	0.5351 ± 0.1247	**0.5419** **±** **0.1240**
BL rMSE	3.1454 ± 0.2630	3.0738 ± 0.3404	3.0246 ± 0.3262	3.1666 ± 0.2885	3.0441 ± 0.3272	**3.0206** **±** **0.3336**
M12 rMSE	3.0863 ± 0.4539	2.9311 ± 0.3865	2.8867 ± 0.3795	3.0925 ± 0.4455	2.9022 ± 0.4169	**2.8690** **±** **0.3839**
M24 rMSE	2.7858 ± 0.3400	2.6819 ± 0.2855	2.6470 ± 0.2954	2.8290 ± 0.3699	2.6632 ± 0.3016	**2.6380** **±** **0.3253**
M36 rMSE	2.3208 ± 0.7000	2.2783 ± 0.4832	2.3415 ± 0.5395	2.3693 ± 0.6535	**2.2978** **±** **0.4541**	2.3546 ± 0.4869

Note that the best results are boldfaced. The superscript symbol “±” indicates that GFL-SGL significantly outperformed that method on that score. Paired *t*-test at a level of 0.05 was used.

## Data Availability

The data used to support the findings of this study can be obtained from the Alzheimer's Disease Neuroimaging Initiative (ADNI) database (http://adni.loni.usc.edu/).
